# Biobehavioral synchrony between three to four months old infants and psychologically healthy mothers during the face-to-face still-face paradigm

**DOI:** 10.1038/s41598-026-65050-1

**Published:** 2026-08-05

**Authors:** Lea Woelflein, I. Brenner, J. Koenig, I. Böhm, M. Eckstein, B. Ditzen, N. Nonnenmacher, C. F. J. Woll-Weber, M. Müller, H. Christiansen, C. Schwenck, R. Steinmayr, L. Wirthwein, M. Kieser, K. Otto, C. Reck, A.-L. Zietlow

**Affiliations:** 1 Faculty of Psychology, Institute of Clinical Psychology and Psychotherapy, Chair of Clinical Child and Adolescent Psychology, Technische Universität Dresden, Chemnitzer Str. 46, Dresden, Germany; 2 Faculty of Medicine and University Hospital Cologne, Department of Child and Adolescent Psychiatry, Psychosomatics and Psychotherapy, University of Cologne, Cologne, Germany; 3 Institute of Medical Psychology, Center for Psychosocial Medicine, Heidelberg University Hospital, Heidelberg, Germany; 4 Clinical Biopsychology and Psychotherapy, Psychological Institute, University of Zurich, Zurich, Switzerland; 5 Deutsches Zentrum für Psychische Gesundheit (DZPG), Berlin, Germany; 6 Clinical Psychology of Childhood and Adolescence, Faculty of Psychology and Educational Sciences, Ludwig-Maximilians-Universität München, Munich, Germany; 7 Clinical Child and Adolescent Psychology and Psychotherapy, Freie Universität Berlin, Berlin, Germany; 8 Department of Clinical Child and Adolescent Psychology, Faculty of Psychology, Philipps University Marburg, Marburg, Germany; 9 Department of Clinical Child and Adolescent Psychology, Faculty of Psychology and Sports Science, Justus-Liebig-University Gießen, Gießen, Germany; 10 Department of Psychology, Faculty of Educational Sciences and Psychology, Technical University Dortmund, Dortmund, Germany; 11 Institute of Medical Biometry, Heidelberg University, Heidelberg, Germany; 12 Department of Social Psychology, Business and Methods, Faculty of Psychology, Philipps University Marburg, Marburg, Germany

**Keywords:** Biobehavioral synchrony, Mother-infant interaction, Physiological synchrony, Heart rate variability, Still-Face paradigm, Maternal bonding, Neuroscience, Physiology, Psychology, Psychology

## Abstract

Human social interaction emerges through coordinated behavior, emotion, and physiology. Biobehavioral synchrony supports stress regulation, attachment, and socio-emotional development, yet its manifestation in psychologically healthy mother-infant dyads remains poorly understood. In particular, little is known about how behavioral and physiological modalities interact and how synchrony varies across contexts. To address this, 94 mother-infant dyads with 3-4-months-old infants participated in the Face-to-Face Still-Face paradigm while cardiac activity was recorded simultaneously in mothers and infants. Physiological synchrony was quantified using cross-correlations of heart rhythm and heart rate variability (HRV) time series, averaged in 10-second windows. We coded behavioral synchrony and assessed bonding via maternal self-report. Behavioral synchrony was higher during play but declined after the stressor, while physiological synchrony was primarily evident during play and lagged 10 s, with maternal responses following infant signals. Low behavioral synchrony corresponded to stronger negative physiological synchrony, indicating that physiological co-regulation persisted despite behavioral misalignment. Supporting theories of early co-regulation, higher behavioral synchrony was linked to fewer bonding difficulties. These findings suggest that low-risk mothers show coordinated physiological fluctuations with their infants, highlighting maternal responsiveness as a key co-regulatory mechanism, providing a framework to identify atypical parent-infant dynamics and guiding parenting interventions that support child development.

**Supplementary Information:**

The online version contains supplementary material available at 10.1038/s41598-026-65050-1.

## Introduction

Human social interaction is inherently reciprocal, involving dynamic coordination of behavior, emotion, and physiological states between individuals. This reciprocity, known as *biobehavioral synchrony*^1^, is particularly crucial in infancy, when caregivers support infants’ stress and emotion regulation before self-regulatory capacities emerge^2^. The Theory of Biobehavioral Synchrony^1^ proposes that such early interpersonal coordination shapes infants’ expectations of social exchange and forms the foundation for attachment, socio-emotional development, and later self-regulation^3–7^. Importantly, synchrony is dynamic and not defined by constant maximum coordination^8^. Natural fluctuations and the ability to re-engage after brief disruptions or mismatches reflect functional interaction and are particularly relevant for developmental outcomes^7^. Characterizing these early synchrony processes is therefore essential for identifying deviations from typical patterns, enabling early detection of developmental risks, and informing parenting-focused interventions.

At the behavioral level, early parent-infant interactions unfold through reciprocal exchanges of gaze, affect, and vocalizations, often described as a synchronized “dance”^7,9^. Through this coordination, dyads develop mutually regulated interaction patterns that shape the quality and stability of their relationship^1^. Parental behaviors such as infant-directed speech activate infants’ physiological systems, creating a bidirectional link between behavior and physiology^1,10^. Situations that disrupt the interaction challenge infants’ emerging regulatory capacities and further elicit active parental co-regulation efforts. These dynamics are commonly investigated using the Face-to-Face Still-Face (FFSF) paradigm^11^, a validated social-stressor task comprising three episodes: emotionally attuned play, a still-face episode where the caregiver becomes unresponsive, and a reunion phase marked by renewed co-regulatory engagement^12^. The still-face episode induces a marked disruption of dyadic interaction. Although engagement typically resumes during the reunion phase, behavioral synchrony remains attenuated relative to the initial play episode^13^. Accordingly, the dyad’s capacity to re-establish coordinated engagement is considered an index of interaction quality^7^.

Physiological coordination, particularly within the autonomic nervous system (ANS), plays a central role to these processes^4^. During the first year postpartum, parasympathetic nervous system (PNS) functioning is pivotal for characterizing infants’ emerging self-regulatory capacities^14,15^ and is typically assessed via heart rate variability (HRV), with elevated HRV levels reflecting enhanced PNS activity^16^. Consistent with models of biobehavioral synchrony positing an interplay between physiology and behavior, research in earliest infancy demonstrates that PNS activity predicts infant social interaction behaviors^17^. Physiological synchrony, defined as correlated fluctuations in HRV between parent and infant, provides a window into dyadic attunement^4^. *Positive synchrony* occurs when HRV changes in the same direction in both partners, (i.e., higher parental HRV is associated with higher infant HRV), whereas *negative synchrony* reflects opposing physiological patterns (i.e., increase in one partner’s HRV corresponds with a decrease in the other’s). These patterns can emerge simultaneously, referred to as concurrent synchrony, or with a temporal lag (i.e., changes in the infant are followed by subsequent changes in the mother, or vice versa), highlighting the dynamic nature of physiological coordination^18^.

As with behavioral synchrony, physiological synchrony likely varies across the FFSF paradigm, as the episodes impose distinct social and regulatory demands. Studies show that physiological synchrony tends to be strongest in tasks requiring high levels of interaction between partners^19,20^. Accordingly, *concurrent synchrony* was shown to be positive during free play, supporting infant emotion regulation^10,21^. During the reunion phase, it shifted negatively, with maternal arousal increasing while infants calm physiologically, reflecting effective maternal soothing behaviors^22^. *Lagged synchrony*, in which one partner’s HRV predicts the other’s subsequent response, remains less studied in the context of mother-infant interactions. Compared to concurrent synchrony, lagged synchrony provides insight into leader-follower dynamics and allows a better understanding of directional influence within the interaction process^18^. There is evidence of both mother-led and infant-led dynamics in healthy dyads, though strength and direction of these effects vary across the FFSF paradigm. Positive mother-to-infant synchrony, where higher maternal HRV predicts subsequent increases in infant HRV, is strongest during the play episode but may decrease or shift negative across subsequent phases^17,23^. During the reunion phase, mother-led synchrony has also been observed as negative, with higher maternal HRV predicting lower infant HRV in the following second, a pattern interpreted as adaptive stress regulation, in which mothers actively lead the regulatory process^24^. Notably, these mother-led patterns were absent in dyads with mothers experiencing postpartum depression or emotional dysregulation^23,24^. Infant-to-mother dynamics appear more stable but vary in direction: positive infant-led effects during reunion occur regardless of maternal mental health^24^ and negative infant-led effects remain consistent across the FFSF paradigm^17^, suggesting that mothers’ following of infant signals and delivery of co-regulatory behaviors may represent a more stable process in early infancy. However, some studies could not find an infant-led dynamic^23^. Together, these findings illustrate that physiological co-regulation is dynamic and context-dependent.

Behavioral and physiological synchrony are theorized to be bidirectionally linked^25,26^, arising from intraindividual loops between behavior and physiology, with these modalities becoming temporarily aligned across partners: For example, changes in the infant’s state may influence maternal physiology, which in turn shapes maternal behavior and feeds back into the infant’s physiology. However, empirical findings remain inconsistent, in part because many studies examine each modality separately or relate synchrony in one modality to individual markers in the other. Evidence from the FFSF paradigm points to context-dependent associations: higher behavioral synchrony has been linked to lower infant and higher maternal HRV during the FFSF paradigm, suggesting increased reliance on maternal co-regulation^6^, whereas higher behavioral synchrony during reunion has also been observed alongside lower maternal HRV, possibly reflecting elevated maternal regulatory effort^27^. Moreover, higher behavioral synchrony during play has been associated with better infant physiological recovery during the reunion episode^28^. Research on physiological synchrony often focuses on one partner’s behavior, overlooking dyadic coordination. For example, positive concurrent HRV synchrony during play has been linked to better infant emotion regulation during reunion^21^, and greater parental physiological responsivity to infant arousal has been associated with faster infant calming^29^. Indeed, one study investigating mother-infant dyads during free play reported that concurrent physiological synchrony increased significantly during episodes of affective and vocal synchrony^10^. In older children, results are mixed: some studies in preschool dyads report a negative association under high-risk conditions, such that positive behavioral synchrony is highest when physiological synchrony is low^30^, whereas other studies spanning preschool to middle childhood do not confirm links between physiological and behavioral synchrony^20,31^. Although studies in infancy rarely examined the link between behavioral and physiological mother-infant synchrony, preliminary evidence at the individual level suggests that the association between infants’ physiological regulation and behavior varies across FFSF episodes, indicating that coupling between physiological and social processes is not uniform^17,32^. This variability may also emerge at the dyadic level. In summary, findings on physiological synchrony between mother and infant, as well as its association with behavioral synchrony, remain limited and heterogeneous.

The heterogeneity of findings may lie, in part, in contextual factors^8^: Meta-analyses indicate that synchrony patterns are shaped by task characteristics and dyadic risk factors, including parental mental health, negative affect and socio-economic status^33–37^. For behavioral synchrony, systematic reviews indicate that sociodemographic risks (e.g., low family income) and familial stressors (e.g., household chaos) are associated with reduced parent-child behavioral synchrony^36^, and suggest diminished mother-infant behavioral synchrony in the context of preterm birth and, albeit with mixed evidence, perinatal depression^35^. With respect to physiological synchrony, a meta-analysis reported a modest positive association in concurrent mother-child HRV synchrony across ages 0.4 to 17 years, accompanied by substantial between-study heterogeneity^34^. Lower HRV synchrony was observed in samples including mothers with psychological symptoms or low socioeconomic status, whereas synchrony did not vary as a function of task challenge or child age^34^.

Taken together, these findings suggest that while contextual and dyadic factors shape parent-infant synchrony, individual parental characteristics may further modulate these patterns and, in turn, be shaped by them. In this context, parental bonding is the multidimensional tie that connects parents to their infant and encompasses cognitive, emotional, behavioral, and neurobiological components^38^. This bond supports parents in meeting caregiving demands and maintaining emotional connection across development. Stronger bonding has been linked to greater maternal sensitivity, emotional responsiveness, and positive interaction behavior^39,40^, all key features of synchronous exchanges. Moreover, higher bonding quality is associated with more positive infant mood, easier temperament, and higher attachment quality^41^. Given the reciprocal nature of parent-infant interaction, synchrony may both support and be influenced by bonding quality, underscoring the need to examine their dynamic interplay. The Theory of Flexible Multimodal Synchrony^8^ builds on this perspective, emphasizing that biobehavioral synchrony is contextually adaptive, shaped by individual differences and relationship variables. Despite its theoretical relevance, the empirical link between synchronous interaction and parental bonding remains largely unexplored.

Drawing on theoretical models of biobehavioral synchrony and prior empirical findings, our hypotheses were as follows: First, we expected mothers and infants to exhibit synchrony at both behavioral (H1) and physiological levels (H2), with synchrony varying across the FFSF phases: high during play, decreasing during still-face, and re-emerging during reunion. Second, based on the strong interdependence of behavioral and physiological processes in early mother-infant interactions and in line with the concept of biobehavioral synchrony, we hypothesized that behavioral and physiological synchrony would be positively associated, with the strength of this association varying across episodes of the FFSF paradigm (H3). Finally, we anticipated that greater behavioral and physiological synchrony would be associated with stronger maternal bonding, reflected in fewer self-reported bonding difficulties (H4).

## Results

Ninety-four mother-infant dyads participated in the FFSF paradigm to examine behavioral and physiological synchrony across the different phases. Participant characteristics are provided in Table 1. Mothers reported low depressive symptoms on the Edinburgh Postnatal Depression Scale (EPDS^42^; *M* = 2.76, *SD* = 2.67); only one scored above the clinical cut-off (*≥* 13;^43^. Given that psychological health was primarily determined through clinical interviews, this participant was retained in the analyses. Overall, the EPDS scores confirmed the sample’s psychological well-being.

**Table 1:** Demographics and descriptive statistics.

	M (*N*)	SD	Min - Max
Maternal age (years)	32.52 (90)	4.02	23–43
Maternal Body Mass Index (BMI)	23.26 (92)	3.09	17.67–32.95
Infant age at FFSF paradigm (months)	3.59 (93)	0.33	2.99–4.87
Infant size at birth (cm)	52.2 (92)	2.84	42–58
Infant weight at birth (grams)	3560.7 (92)	493.12	2000–4999
PBQ-16	7.04 (92)	4.21	0–18
EPDS	2.76 (91)	2.67	0–13
Behavioral Synchrony (across phases)	3.57 (87)	0.70	1.83-5
Behavioral Synchrony (play phase)	3.71 (87)	0.71	1.83-5
Behavioral Synchrony (reunion phase)	3.43 (87)	0.82	1.5-5
Physiological Synchrony (RMSSD)	-0.01 (57)	0.28	-0.77-0.87
Physiological Synchrony (IBI)	-0.03 (57)	0.33	-0.93-0.92
	*N (%)*		
Infant gender			
Female	43 (46.2%)		
Maternal education			
No degree	1 (0.1%)		
High or low secondary qualification	34 (38.6%)		
University entrance qualification	5 (5.7%)		
University degree	48 (54.5%)		
Partnership status			
Married	74 (80.4%)		
Permanent partnership	18 (19.6%)		
Household income			
0–1000 €	1 (1.1%)		
1000–2000€	6 (6.5%)		
2000–3000€	6 (6.5%)		
3000–5000€	45 (48.9%)		
5000€ and more	34 (37.0%)		

PBQ-16 = Postpartum Bonding Questionnaire Revised; EPDS = Edinburgh Postnatal Depression Scale; RMSSD = square root of the mean of the sum of all squared differences between adjacent RR intervals; IBI = inter-beat interval; Physiological synchrony values represent mean cross-correlations aggregated across all examined lags.

### Phase differences in behavioral mother-infant synchrony

For behavioral synchrony (Analysis 1), a linear mixed-effects model revealed that behavioral synchrony decreased from the play (*b* = 3.71, *SE* = 0.08) to the reunion phase (*b* = -0.29, SE = 0.07, *p* < .001, ß = − 0.37; Supplementary Table S1).

### Physiological synchrony across the phases of the FFSF paradigm

#### Concurrent physiological synchrony

To examine phase-related differences in physiological synchrony, multilevel models predicted concurrent cross-correlations of inter-beat intervals (IBIs) and log-transformed RMSSD (square root of the mean of the sum of squared differences between adjacent RR intervals) between mothers and infants (Analysis 2a). No significant phase effects emerged for either IBI (all *p*_s_ > 0.750) or RMSSD (all *p*_*s*_ > 0.738) cross-correlations (see Supplementary Table S2, IBI model; Supplementary Table S3, RMSSD model). Analysis of estimated marginal means for each phase revealed that cross-correlations for both IBI and RMSSD did not differ significantly from zero (all *p*_*s*_ > 0.342), indicating no evidence of concurrent physiological synchrony. Sensitivity analyses indicated that effect sizes of β = 0.25 (IBI) and β = 0.20 (RMSSD) would be required to achieve adequate power (85–93%) to detect phase-related differences. Smaller effects would likely remain undetected, suggesting that the current study may have been underpowered to identify small phase-specific variations in physiological synchrony.

#### Lagged physiological synchrony

**Fig. 1:**
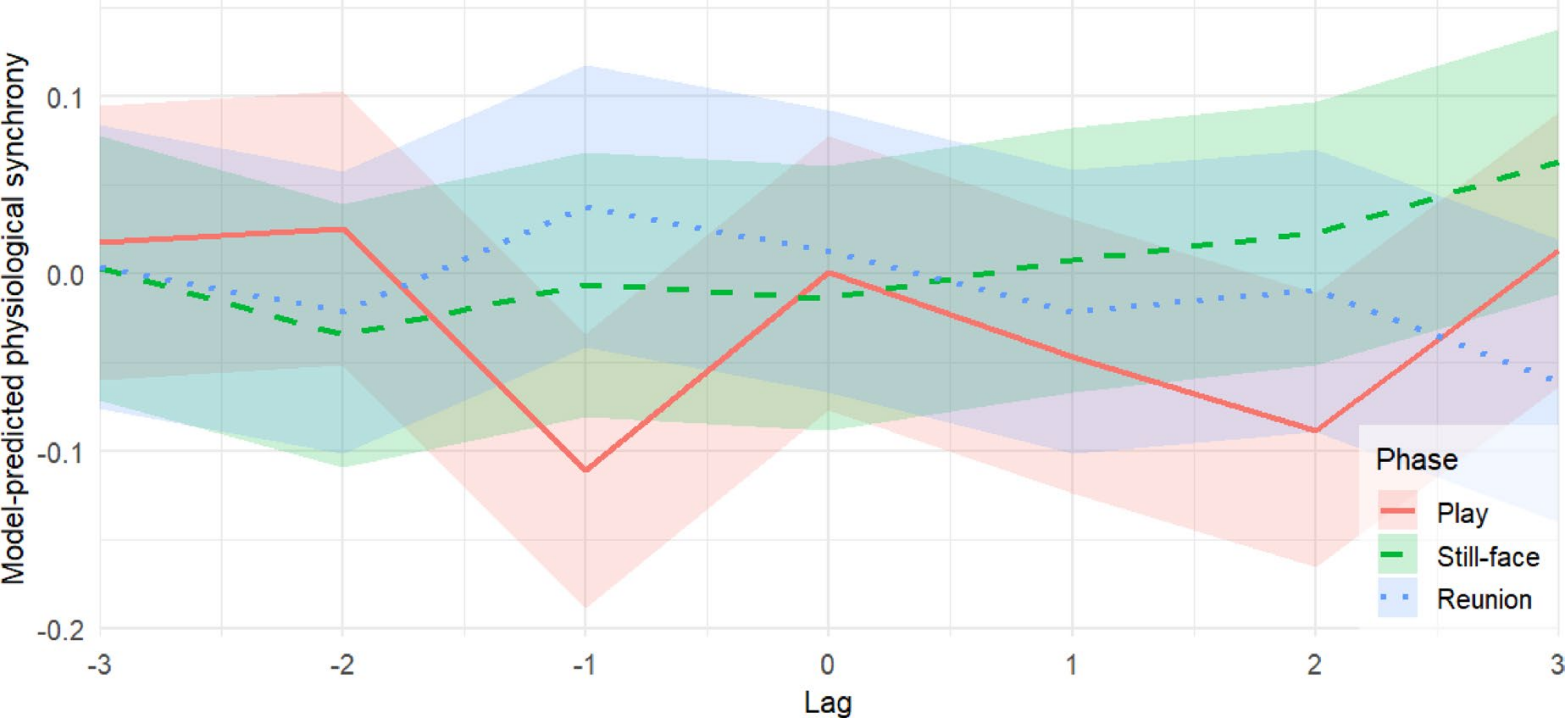
Physiological synchrony between mother and infant operationalized as cross-correlation coefficients of log-transformed RMSSD, for each lag and displayed separately for each phase of the FFSF paradigm. Lags represent 10-second segment shifts, with negative values indicating the infant leads and positive values the mother leads (lag 3 = 30 s). Shaded areas represent the 95% confidence intervals.

Building on the assumption of early co-regulatory processes within mother-infant interactions, we tested whether changes in one partner’s physiological state predicted subsequent changes in the other (lagged synchrony, Analysis 2b). For IBI cross-correlations, no significant effects of temporal lag, phase or their interaction were observed (all *p*_*s*_ > 0.284), indicating no lag- or phase-related differences (see Supplementary Table S4 for full model results). For RMSSD cross-correlations (Fig. 1), a significant lag effect emerged: synchrony at lag − 1 was lower than at the reference lag 0 (*b* = -0.11, *SE* = 0.06, *p* = .044, ß = − 0.40), suggesting that infant HRV changes preceded maternal HRV changes by approximately 10 s - indicating a pattern in which the infant leads and the mother follows. RMSSD synchrony did not vary across FFSF paradigm phases, and no lag × phase interaction was found (see Supplementary Table S5 for full model results). Estimated marginal means were tested against zero using Bonferroni correction across lags within each phase. Only in the play phase did RMSSD synchrony at lag − 1 differ significantly from zero (*M* = -0.11, *SE* = 0.04, *p* = .035). No other lag showed significant synchrony.

#### Does the observed physiological mother-infant synchrony exceed chance level?

To provide an initial check that HRV synchrony exceeds chance, we created pseudo-dyads by randomly reassigning each mother’s data to an unrelated infant in a single iteration. Cross-correlations in these surrogate pairs showed no significant lagged effects for RMSSD (Analysis 2b), indicating that observed synchrony reflects genuine mother-infant physiological coordination rather than coincidental alignment (see Supplementary Table S6 for full model results).

### Association between physiological and behavioral synchrony

**Fig. 2:**
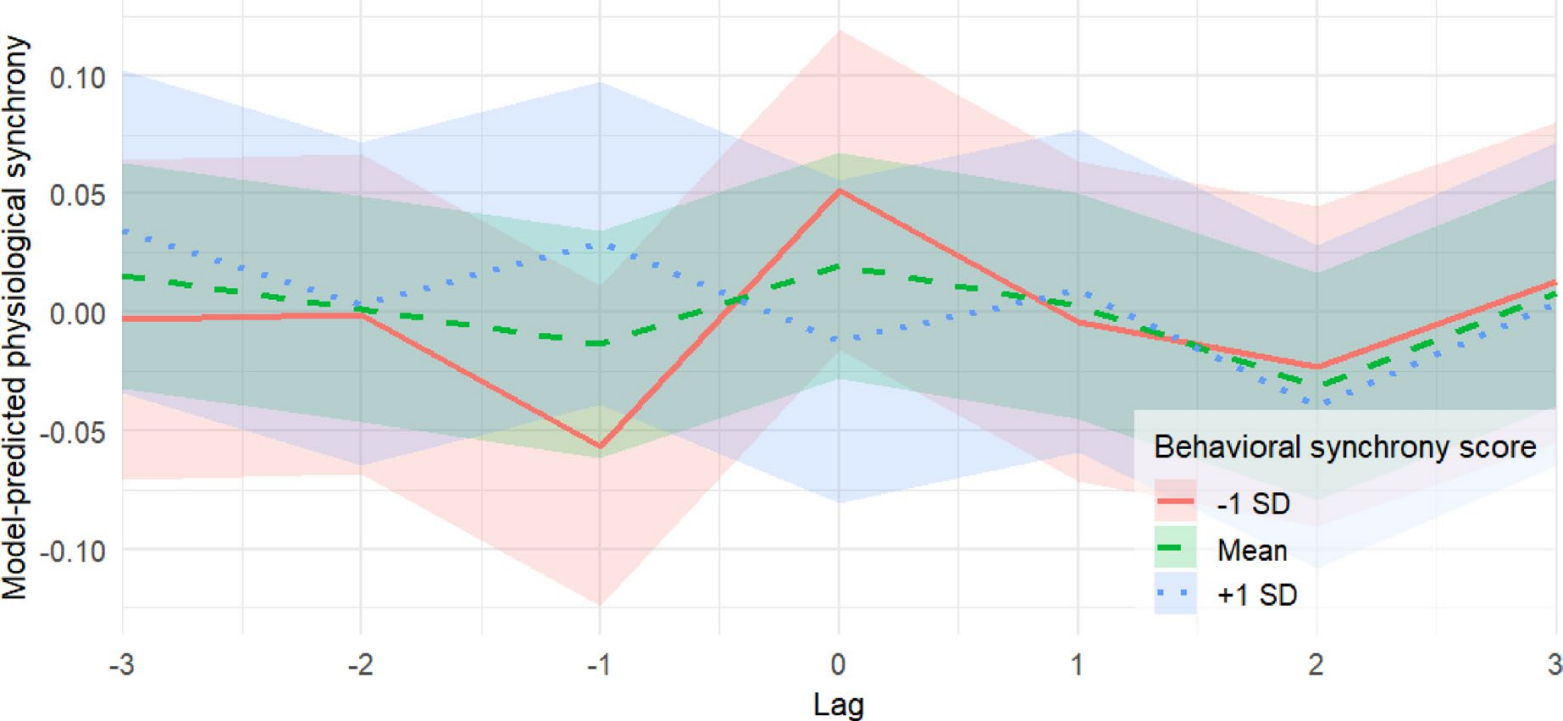
Physiological synchrony across all phases of the FFSF paradigm, operationalized as cross-correlation coefficients of log-transformed RMSSD across lags of 10-second segments. Negative lags indicate the infant leads, positive lags the mother leads (lag 3 = 30 s). Results are displayed separately for low (− 1 *SD*), average (*M*), and high (+ 1 *SD*) behavioral synchrony. Shaded areas represent the 95% confidence intervals.

Building on the observed lagged physiological synchrony, we examined the association between behavioral and physiological synchrony (Analysis 3). For RMSSD cross-correlations, lag − 1 showed significantly lower synchrony (*b* = -0.48, *SE* = 0.20, *p* = .019, ß = − 0.12). Importantly, a significant interaction between behavioral synchrony and lag − 1 emerged (*b* = 0.12, *SE* = 0.06, *p* = .028, ß = 0.27), indicating that higher behavioral synchrony attenuated negative lagged physiological synchrony, whereas lower behavioral synchrony was associated with more negative RMSSD cross-correlations (Fig. 2; see Supplementary Table S7). However, testing the estimated marginal means revealed no significant deviations from zero (all *p*_*s*_ >0.678). No significant effects were observed for IBI cross-correlations (see Supplementary Table S8 for model results).

**Fig. 3:**
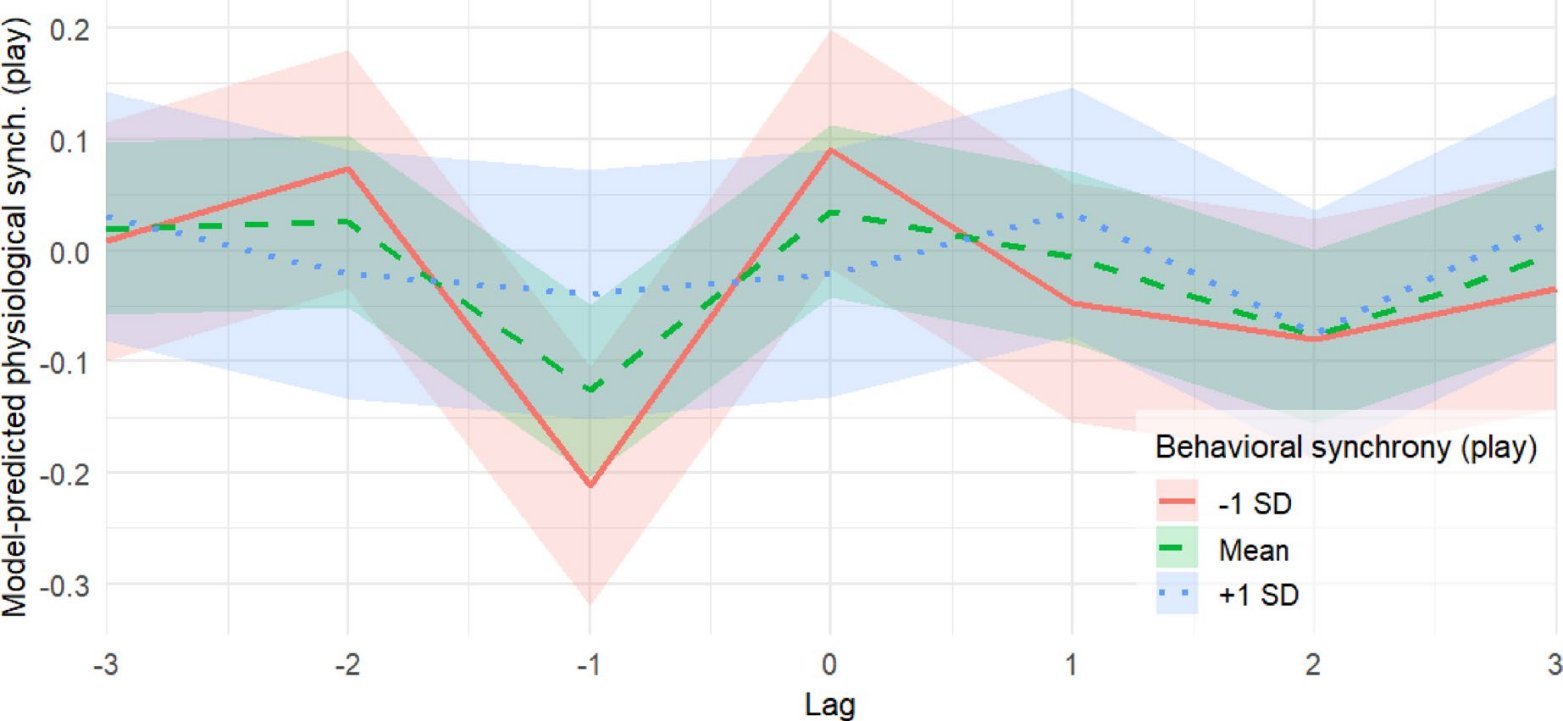
Physiological synchrony during the play phase, operationalized as cross-correlation coefficients of log-transformed RMSSD across lags of 10-second segments. Negative lags indicate the infant leads, positive lags the mother leads (lag 3 = 30 s). Results are displayed separately for low (− 1 *SD*), average (*M*), and high (+ 1 *SD*) behavioral synchrony for the play phase only. Shaded areas represent the 95% confidence intervals.

Given that behavioral synchrony was measured only during play and reunion phases, we conducted an exploratory follow-up analysis using phase-specific scores. No significant effects emerged during the reunion phase (see Supplementary Table S9). During play, however, lag − 1 significantly predicted RMSSD synchrony (*b* = -0.99, *SE* = 0.34, *p* = .004, ß = − 0.65), indicating more negative physiological synchrony at this lag compared to lag 0. Moreover, a significant interaction with play-specific behavioral synchrony was observed (*b* = 0.22, *SE* = 0.09, *p* = .016, ß = 0.50), such that lower behavioral synchrony was associated with stronger negative RMSSD cross-correlations at lag − 1 (Fig. 3; see Supplementary Table S10). Estimated marginal means of RMSSD synchrony were computed at the mean and ± 1 SD of behavioral synchrony across all seven lags. Only lag − 1 showed significant deviations from zero: negative RMSSD synchrony was significantly different from zero for lower-than-average (*M* = − 0.22, *SE* = 0.06, *p* = .001) and at average behavioral synchrony (*M* = − 0.14, *SE* = 0.04, *p* = .007), but not for higher-than-average synchrony (*M* = − 0.05, *SE* = 0.06, *p* = 1).

### Biobehavioral mother-infant synchrony and its association with maternal bonding

Since biobehavioral synchrony has been proposed as a foundation for mother-infant affiliation and attachment^4^, we explored its association with maternal bonding. This hypothesis was included in the preregistration without directional specification, and the corresponding analyses are therefore exploratory in nature. For IBI-based synchrony, neither maternal bonding (*b* = 0.01, *SE* = 0.00, *p* = .118, ß = 0.07) nor interactions reached significance (see Supplementary Table S11). Similarly, the RMSSD model showed no significant effect of maternal bonding difficulties (*b* = -0.00, *SE* = 0.00, *p* = .620, ß = − 0.02). Importantly, the previously observed lag − 1 effects persisted: lag − 1 (*b* = -0.48, *SE* = 0.20, *p* = .019, ß = − 0.12) and its interaction with behavioral synchrony (*b* = 0.12, *SE* = 0.06, *p* = .028, ß = 0.27) remained significant in predicting physiological synchrony (see Supplementary Table S12). Bivariate correlations for physiological synchrony were non-significant (Supplementary Table S13), while a small negative correlation emerged between bonding difficulties and behavioral synchrony (*r* = − .14, *p* < .001, 95% CI [-0.20, -0.08]).

As a robustness check, analyses were repeated using Fisher z-transformed cross-correlation coefficients. Results were consistent with those obtained from the original coefficients and did not differ in statistical significance (Supplementary Tables S14–S24).

## Discussion

Early biobehavioral synchrony is foundational to attachment and interpersonal behavior^4^, yet its strength, direction, and contextual determinants remain incompletely understood. To our knowledge, this is the first study to jointly examine behavioral and physiological synchrony across the episodes of the FFSF paradigm in a clinically well-characterized, low-risk sample of 3–4 month old infants – a developmental period characterized by emerging face-to-face engagement and rapid parasympathetic maturation^1,44,45^.

First, we found that behavioral synchrony was higher during the play episode than during reunion following maternal disengagement, suggesting that synchrony may be more easily established in the absence of prior disruption. Second, physiological synchrony emerged with a temporal lag, with maternal HRV following infant HRV by approximately 10 s. Accounting for this temporal lag, lower behavioral synchrony was associated with stronger negative physiological synchrony, particularly during play. Finally, behavioral synchrony was higher in dyads in which mothers reported fewer bonding difficulties, highlighting a potential link between early interactive alignment and parent-infant relationship quality.

### Higher behavioral synchrony during the play episode

Consistent with prior research^13,20,33^, interaction context affected behavioral synchrony: the “still-face”-episode temporarily disrupted dyadic exchanges, and synchrony remained reduced during reunion compared with the play episode, likely reflecting the time required to re-establish mutual engagement following maternal disengagement. This pattern suggests that behavioral synchrony is highly sensitive to changes in interaction context^12^.

### Evidence for lagged but not concurrent physiological synchrony in early infancy

While concurrent physiological synchrony has been documented in same-age or slightly older infants - for example, positive synchrony during play at 3–6 months^10,21^ and negative synchrony during reunion at 5 months^22^- we did not observe concurrent synchrony in our younger sample (3–4 months). This may indicate that simultaneous physiological coupling is not yet fully established at this developmental stage. Instead, early caregiver-infant interactions may primarily rely on lagged, caregiver-driven processes, in which caregivers tend to adjust their physiological responses to preceding infant signals, reflecting infants’ high dependence on external regulation. As children develop greater self-regulatory capacities, synchrony may shift toward more reciprocal and simultaneous forms of coordination^30^.

Consistent with this interpretation, we observed infant-led lagged physiological synchrony characterized by a negative association, such that maternal HRV increased when infant HRV decreased, and vice versa. This pattern may suggest a compensatory regulatory process in which mothers respond to infant dysregulation by up- or downregulating their own parasympathetic activity to buffer and stabilize the infant, supporting co-regulation rather than simple mirroring. These findings align with theoretical models of early co-regulation^4^, suggesting that mothers are responsive to infants’ signals, with behavioral responsiveness both supporting and being supported by autonomic co-regulation. They are also consistent with reports of negative lagged physiological synchrony in dyads with 9-month-old infants^17^. However, findings across studies remain mixed, with reports of both positive infant-led synchrony in 5-month-olds^24^, and absent infant-led effects in 7-month-old infants^23^. The mixed findings may reflect a combination of developmental differences across infant age and methodological variation, including differences in interaction paradigms and analytical approaches.

Contrary to our hypothesis, lagged physiological synchrony did not vary across episodes of the FFSF paradigm. This finding aligns with prior work suggesting that infant-led negative synchrony may be stable across social contexts^17^. Its presence during the still-face episode suggests that such coupling may persist under stress, potentially reflecting a stable regulatory mechanism rather than context-dependent coordination^24^. Although we did not observe phase-specific differences, synchrony was primarily driven by the play episode, suggesting that this context is particularly conducive to mother-infant coordination. This aligns with evidence that synchrony is strongest in contexts permitting high reciprocal engagement^10,19,20^. However, its functional significance – including potential links to overall relationship quality – remains unclear and a full characterization of this dynamic across stress conditions and interaction partners is still pending.

### Behavioral and physiological synchrony as potential buffering mechanisms

Consistent with models of biobehavioral synchrony^1,8^, synchrony is a dynamic process fluctuating between coordinated and uncoordinated states. Accordingly, we aimed to identify conditions under which behavioral and physiological synchrony align. An association emerged during the play episode when maternal HRV lagged infant HRV by one 10-second interval. Specifically, lower behavioral synchrony was linked to stronger negative physiological synchrony, potentially reflecting compensatory autonomic co-regulation when behavioral alignment was reduced. Although phase effects were not significant overall, this pattern was specific to the play episode, indicating context sensitivity. This finding aligns with evidence that coupling between infant behavior and physiology emerges primarily during active caregiver engagement^17^. Studies in preschool- and school-aged children have also reported a negative association, with positive behavioral synchrony being highest when physiological synchrony is low^30^. However, our findings differ from previous reports of either stronger physiological synchrony during periods of high behavioral synchrony^10^ or no association between these modalities^20,31^. Such discrepancies may reflect developmental and contextual factors. Behavioral and physiological processes may be more tightly coupled in early infancy, when infants rely heavily on caregiver responsiveness^1^, whereas the emergence of self-regulatory capacities and increasing social complexity may reduce coupling between these modalities later in development.

### Divergent associations of behavioral and physiological synchrony with bonding

In line with established theories of interaction, which posit that interpersonal synchrony supports the development of affiliative bonds^1^, higher behavioral synchrony was associated with fewer maternal bonding difficulties, consistent with evidence linking stronger bonding to higher-quality interactions^39,40^. Importantly, the present findings do not allow conclusions regarding the direction of this association. No comparable association was observed for physiological synchrony, possibly because behavioral exchanges are directly observable and may therefore influence caregivers’ perceptions of bonding more strongly than unconscious physiological alignment. Similarly, behavioral synchrony was related to several demographic and relational factors, indicating sensitivity to context-dependent and observable interaction patterns, whereas physiological synchrony showed no such associations, potentially reflecting more stable autonomic co-regulatory processes that are less dependent on contextual influences (Supplementary Table S13). These differential associations are theoretically informative, reflecting functional differentiation or compensatory processes, suggesting that behavioral and physiological synchrony capture partly distinct aspects of dyadic functioning. This interpretation is consistent with theoretical accounts of biobehavioral synchrony, which emphasize that coordination unfolds across multiple, partly dissociable systems that may not necessarily operate in parallel^1^. Considering both convergence and divergence between modalities may therefore provide a more comprehensive understanding of early dyadic regulation.

### Limitations

Several limitations warrant consideration for future research. First, behavioral and physiological synchrony were assessed at different temporal resolutions, with HRV cross-correlations based on 10-second intervals and behavioral synchrony coded across 2-minute phases. Moreover, physiological synchrony spanned a continuum from positive to negative values, whereas behavioral coding captured the degree of interactional coordination. These differences may have attenuated cross-modal associations. Moreover, macro-level behavioral coding may be less sensitive to moment-to-moment fluctuations, such as recovery dynamics during the reunion episode, potentially obscuring processes unfolding at shorter timescales. Second, the choice of 10-second HRV epochs may have influenced observed patterns. Prior work suggests that shorter epochs (e.g., 1-s) capture both positive and negative infant- and mother-led dynamics^17,24^, whereas longer epochs (e.g., 30 s) tend to show primarily mother-led temporal effects^23^. Accordingly, interaction dynamics occurring at different timescales may thus not have been fully captured in the present analyses, potentially contributing to the absence of mother-led temporal effects. In addition, cross-correlations based on short time series may be unstable, although RMSSD provides reliable HRV estimates in 10-second segments^46^. Further, we did not explicitly control for autocorrelation, which could produce apparent cross-correlations unrelated to genuine interpersonal coupling. Lagged estimates within dyads are inherently interdependent, and while our random-intercept models account for between-dyad variability, they do not fully capture within-dyad dependencies across lags. However, the available data structure did not allow for a more complex modelling approach that would adequately model these dependencies. Although methods such as first‑differencing could address autocorrelation, analytical choices in HRV time series can substantially affect derived physiological indices, and care should be taken not to remove meaningful dynamics^47^. To address concerns regarding inflated Type I error, we implemented pseudo-dyad control analyses, largely preserving the original data structure. Third, sensitivity analyses suggested that the study may have been underpowered to detect phase-related differences in physiological synchrony. Indeed, one study reported phase-dependent changes in mother-infant physiological synchrony, with synchrony shifting from positive to negative across the FFSF paradigm in dyads characterized by low maternal emotional dysregulation^23^. Accordingly, the absence of phase effects in the present study should be interpreted cautiously, as null findings may reflect limited statistical power rather than the absence of coupling. Finally, we did not account for individual differences in infants’ responses to the Still-Face episode^12,14^. In particular, higher levels of infant negative affect have been associated with reduced synchrony^37^, and individual differences in infant physiological reactivity may likewise shape synchrony patterns^14,48^. Exploratory analyses indicate the expected increase in infant negative affect from play to reunion, alongside substantial inter-individual differences, potentially influencing our findings regarding the reunion episode (see Supplementary Table S25 for descriptive statistics; Supplementary Table S26 for model results).

### Future directions

Beyond addressing the limitations outlined above, the present findings provide a foundation for advancing the understanding of biobehavioral synchrony in early mother-infant interactions. Several avenues for future research emerge from our results: Future research using micro-analytic behavioral coding could clarify whether behavioral and physiological synchrony persist or shift at finer temporal resolution. This would enable distinguishing synchronous and non-synchronous moments at the behavioral level and examining how physiological synchrony emerges within them, providing precise insight into the coordination of early interactions^10^. This is particularly relevant for understanding how associations between modalities vary across contexts, such as within different phases of the FFSF paradigm.

At the same time, further methodological work is needed to strengthen the comparability and interpretation of synchrony findings. Given the heterogeneity across studies^17,23,24^, future research should systematically examine how epoch length, handling of autocorrelation, and analytic strategies jointly shape estimates of lagged physiological synchrony and directional effects. In addition, future studies should compare observed effects against a stable null distribution based on multiple randomizations to provide a more robust assessment of chance-level synchrony.

Finally, although we characterized biobehavioral synchrony in healthy mother-infant dyads, it remains unclear which synchrony patterns are most beneficial for child development. Our findings provide a reference point for future studies examining additional contextual and individual factors, including parental clinical risk^34,49^, infant negative affect and physiological reactivity^14,37,48^, which may moderate synchrony processes but remain insufficiently understood. Longitudinal research is needed to determine which forms of synchrony support developmental outcomes, how these patterns evolve as children gain autonomy, and whether different modalities of synchrony differentially predict later outcomes, as suggested by their distinct associations with maternal bonding^3^. Future research should also extend beyond mother-infant dyads to include secondary caregivers in order to assess the generalizability of these findings across caregiving contexts.

## Conclusion

In conclusion, our findings highlight the dynamic, time-sensitive, and bidirectional nature of mother-infant interactions, suggesting that maternal physiological co-regulation may unfold in response to infant signals. Accounting for temporal dynamics revealed that behavioral and physiological synchrony are related under specific contextual conditions, pointing to complementary or buffering functions of these two modalities. Rather than reflecting a single underlying process, behavioral and physiological synchrony appear to capture distinct yet interconnected aspects of early parent-infant interaction that may have different developmental implications. Together, these findings underscore the value of multi-level approaches for understanding the complexity of early social interactions.

## Methods

### Design and procedure

The two studies from which the present sample was drawn were both conducted in accordance with the current guidelines of the World Medical Association (revised Declaration of Helsinki). Study 1 (COMPARE interaction study) was approved by the local ethics committees at the Department of Psychology, Ludwig-Maximilians-University Munich (approval no. 74_Reck_b), and Medical Faculty Heidelberg (no. S-446/2017). The study protocol was preregistered^50^. Study 2 (NeMo study) conducted at Heidelberg University, was approved by Medical Faculty Heidelberg’s ethics committee (no. S-450/2017), and its design and hypotheses were preregistered^51^. Data collection took place at both sites (Heidelberg and Munich) under standardized and identical laboratory conditions between 2018 and 2022. The present study’s design and hypotheses were preregistered (https://aspredicted.org/kv8wx.pdf).

Participants were recruited through both online platforms and printed flyers distributed via various sources, including midwives, gynecologists, and registration offices at our two study sites, Munich and Heidelberg. Following the initial contact, a telephone screening interview was conducted 4 to 10 weeks postpartum to assess preliminary exclusion criteria. Caregivers were provided with detailed explanations of the study procedures, and written informed consent was obtained for their participation. During the laboratory visit (12–16 weeks postpartum), a structured interview was administered to ensure the absence of any maternal current or past mental illness as defined by the DSM-5^52^. Furthermore, mother-infant interaction was assessed in a laboratory setting. The infant was secured in a booster seat and placed on a table face-to-face with the mother. Each mother was given a standardized instruction of the FFSF paradigm^11^ directly before the interaction. Mothers were instructed to engage in a natural interaction with their infants for a duration of two minutes. Following the initial play phase, mothers were instructed to avert their gaze for ten seconds in response to a signal. After this period, they were required to turn back to the infant with a neutral expression and without touching the infant, marking the beginning of the still-face episode, which also consisted of two minutes. Subsequently, mothers received a signal to re-engage with their infants for another two minutes.

Participants completed online questionnaires and forms on demographics or further factors known to influence HRV values (e.g., weight, size) following the laboratory visit.

### Participants

The study sample was drawn from two longitudinal studies with matching inclusion criteria, aligned laboratory visit time points, and identical paradigm procedures relevant to the current study. In line with the inclusion criteria defined for the control groups in the original study protocols^50,51^, participating infants were required to be full-term (≥ 37 weeks of gestation), singletons, free of congenital abnormalities and confirmed physical or developmental disorders, and have Apgar scores ≥ 9 at ten minutes postpartum. Mothers were required to have no severe somatic illnesses, no current or lifetime mental disorder diagnoses, and no history of psychotherapy or more than seven therapeutic counseling sessions at any point in their lives. The total sample of the present study consisted of *N* = 94 mother–infant dyads. Maternal age at birth ranged from 23 to 43 years (*M* = 32.52 years, *SD* = 4.02 years), and infants were aged from 2.99 to 4.87 months (*M* = 3.59 months, *SD* = 0.33 months).

### Measures and instruments

#### Clinical interview

In order to characterize the risk status of the sample, maternal psychopathology was surveyed in detail using the DSM-5 revised version of the Diagnostic Interview in Mental Disorders^53,54^.

#### Depressive symptoms

The German version of the EPDS^42^ was used to assess maternal depressiveness via self-report over the past seven days as an additional control measure for mood. The scale consists of ten items (e.g., “I have been so unhappy that I have had difficulty sleeping”) and responses are made on a four-point Likert scale ranging from 0 to 3. The total score is calculated by summing the individual item scores and can range from 0 to 30. Higher values indicate higher depressiveness. A cut-off value of ≥ 13 is recommended for higher specificity and to detect higher symptom levels^43^. The scale demonstrated acceptable reliability (*α* = 0.74; 95% CI [0.66, 0.81]).

#### Maternal bonding

Maternal bonding was measured with the 16-item Postpartum Bonding Questionnaire (PBQ-16;^55^. The PBQ-16 is a standardized screening instrument for assessing self-reported maternal bonding difficulties. The 16 items (e.g., “I feel distant from my baby”) are rated on a six-point scale from “always” to “never” with a numeric maximum of five. Higher scores on this questionnaire indicate more impaired maternal bonding. The PBQ-16 showed good reliability (*α* = 0.80; 95% CI [0.74, 0.86]).

#### Behavioral synchrony

Mother-infant interaction was recorded from multiple camera angles to allow for the coding of interaction behaviors from both the mother and the infant. Four independent coders, blind to the study hypotheses, rated the mother-infant interactions using the Coding Interactive Behavior manual (CIB;^56^. This is a globally used and reliable instrument for coding interactions and observed behaviors of children and their parents. Raters had successfully completed several days of training with Professor Dr. Ruth Feldman (Reichman University, Herzliya, Israel and Yale University, New Haven, USA), followed by reliability training. The CIB consists of 43 different scales, all of which are assessed on a five-point scale (1 = minimum and 5 = maximum level of each behavior). Of these, 22 scales relate to adult behavior, 16 relate to child behavior, and 5 scales are dyadic, meaning they refer to the interaction between mother and child^56^. Ratings were conducted for both the play and reunion phases of the FFSF paradigm, each lasting two minutes. Three dyadic CIB scales (Dyadic Reciprocity, Fluency, and Adaptation-Regulation) were averaged to create a composite *Behavioral Synchrony Score*, due to their proximity to the concept of behavioral synchrony. The scales were strongly intercorrelated (play: *r* = .68-0.89, all *p*_*s*_ < 0.001; reunion: *r* = .61-0.90, all *p*_*s*_ < 0.001), supporting their aggregation into a composite score. Internal consistency was excellent (play: Cronbach’s α = 0.91; 95% CI [0.88, 0.94]; reunion: Cronbach’s α = 0.89; 95%-CI [0.85, 0.93]). The magnitude of the intercorrelations and the high internal consistencies indicate that the three scales reflect a common underlying construct, justifying their aggregation into a behavioral synchrony composite, while still capturing conceptually distinct aspects of dyadic behavior. This means that at the behavioral level, synchrony was defined as a mutual and coordinated exchange between two interaction partners (dyadic reciprocity), marked by their ability to flexibly adjust behavior and emotional tone in response to one another (adaptation-regulation), and characterized by a smooth, uninterrupted flow (fluency). For reasons of methodological parsimony, these scores were averaged across the play and reunion phases to yield a single behavioral synchrony score for the entire paradigm. This approach was further supported by a statistically significant positive association between behavioral synchrony in the two phases (*r* = .66, 95% CI [0.53, 0.77], *p* < .001) and by strong internal consistency (Cronbach’s α = 0.79; 95% CI [0.69, 0.86]). 18% of the interactions were double coded. Interrater reliability for the behavioral synchrony scores was assessed using the intraclass correlation coefficient (ICC1k, one-way random, absolute agreement) for the average of two randomly selected raters per video. Reliability was moderate across phases, with ICC = 0.73 (95% CI [0.45, 0.89], *p* < .001) for the play phase, ICC = 0.71 (95% CI [0.40, 0.88], *p* < .001) for the reunion phase, and ICC = 0.61 (95% CI [0.19, 0.84], *p* = .005) for the behavioral synchrony score across the paradigm.

#### Physiological synchrony

For each dyad, electrocardiographic data were collected using the EcgMove 4 device (Movisens© GmbH, Karlsruhe, Germany) at a sampling rate of 1024 Hz. Self-adhesive Ag/AgCl electrodes were placed on the chest of both mother and infant by the researcher prior to the start of the interaction paradigm. ECG signals were recorded continuously throughout the FFSF paradigm.

UnisensViewer© (Version 1.12.38.0) was used to cut ECG data with millisecond accuracy based on start and end times of the FFSF Paradigm (Supplementary Fig. S1). We performed a first quality check of the ECG data, evaluating the presence of at least one QRS complex in a 10-second segment, continuous detectability of R-peaks, any prolonged signal dropouts or artifacts, and the plausibility of RR intervals for each participant. R-peak detection and artifact correction of ECG data were conducted in Kubios HRVPremium© (3.1.0). Incorrectly or unidentified R-waves were corrected manually. Artifacts or extended periods of signal distortion led to missing R-peaks, resulting in disproportionately large IBIs. These segments were automatically excluded during further processing based on the criterion of physiologically implausible IBIs.

Corrected IBI time series were exported for later segmentation according to the defined phases of the FFSF paradigm and calculation in STATA SE 18. For artifact correction, we initially excluded physiologically implausible IBI values, specifically those less than 300 ms or greater than 1500 ms^57^, resulting in the removal of 0.68% of all IBIs in our sample. Second, intra-individual artifact correction was applied using each participant’s 25th, 50th, and 75th percentiles. Values beyond Q1–3×IQR or Q3 + 3×IQR were considered extreme, leading to the removal of an additional 0.82% of all IBIs.

For the present analyses, each phase of the FFSF paradigm was divided into 12 segments of 10 s each, following recommendations for microanalytic approaches^46^. This interval length was selected to balance temporal resolution with data stability, allowing detection of concordant changes in mother-infant IBIs while minimizing noise from single-second fluctuations. The following physiological parameters were then calculated as mean values for each 10-second segment: IBIs, which correspond to heart rate (HR) in beats per minute (bpm) and RMSSD. We deviated from the preregistration by using IBI instead of HR, as IBI provides greater temporal accuracy, particularly relevant for lagged synchrony analyses. RMSSD (in milliseconds) has proven to be a reliable time-domain measure of PNS influences^16,58^.

To calculate physiological synchrony between mother and infant, we computed the cross-correlation coefficients (using Stata SE 18, StataCorp LP, College Station, TX, US) for 10-second segments, separately for raw IBI data and RMSSD values. Due to the skewed distribution, the RMSSD values were log-transformed prior to analysis^59^. Phase-specific lagged cross-correlations were computed for 7 lags (ranging from − 3 to 3). Each lag represents a shift of one 10-second RMSSD segment; for example, a lag of 1 corresponds to a 10-second shift. A negative lag indicates that the first signal (infant) precedes the second (mother), while a positive lag means that the first signal (infant) follows the second in time (mother).

Prior research has noted that HRV synchrony may not necessarily exceed chance levels, as it can arise from parallel responses to shared contextual stimuli or common environmental influences^26^. To examine whether the observed synchrony exceeded chance-level co-fluctuations, we conducted a pseudo-dyad analysis. Physiological time series were randomly reassigned between unrelated mothers and infants, thereby generating randomized mother-infant pairs while preserving the temporal structure of each individual’s data. This reassignment was performed once by shuffling participant IDs and pairing each mother with a randomly selected infant from another dyad. Cross-correlations of IBI and RMSSD were then computed for these randomized pairs using the same procedure as described above. The resulting synchrony estimates from pseudo-dyads served as a null comparison to evaluate whether synchrony observed in real dyads exceeded levels expected from coincidental co-fluctuations or autocorrelated physiological signals. Randomized pairs could not be generated for behavioral synchrony because the codes represent dyadic interaction, making recombination across dyads unfeasible.

### Statistical analyses

Analyses were performed using R (Version 4.4.0;^60^. To account for the hierarchical structure of our data, we tested our hypotheses using multilevel modeling, as recommended in the literature on caregiver-child synchrony^3^. These models were estimated using the *lmer* function from the R package *lme4* (version 1.1–35.3), with restricted maximum likelihood (REML) estimation as the default. For handling missing data, we relied on the default approach (listwise deletion) implemented in the *lmer* function.

#### Missing data

The final sample consisted of *N* = 94 dyads who provided data on either behavioral or physiological synchrony, the primary outcomes of interest. Behavioral synchrony was coded for 87 dyads; seven videos could not be coded due to missing audio. For ECG data, the most common reason for missing data was failure to record or store the ECG signal (*n* = 19). In one dyad, the recorded ECG did not overlap with the protocolled interaction period. During preprocessing, 10 additional ECG recordings were excluded due to excessive artifacts (e.g., no detectable QRS complexes). After preprocessing, ECG data were available for both interaction partners in 74 dyads, for only one partner in nine dyads, and for neither partner in 11 dyads, resulting in *n* = 157 individual ECG recordings. For analyses of physiological synchrony using cross-correlations, synchrony indices were calculated only when phase- and outcome-specific data completeness was available for both interaction partners (i.e., 12 valid data segments per partner within a given episode of the FFSF paradigm). Accordingly, IBI and RMSSD cross-correlations could be computed for *n* = 57 dyads. Of these, *n* = 47 dyads provided cross-correlations for all 21 lag-by-phase combinations (7 lags × 3 phases). This resulted in *N* = 50 dyads with data available for both behavioral and physiological synchrony. Of these, *N* = 48 dyads also had questionnaire data on maternal bonding. There were no statistically significant differences between dyads with physiological synchrony data and those without in any of the following variables: maternal and infant age, infant sex, maternal BMI, infant size and weight, EPDS, PBQ-16, behavioral synchrony during play and reunion episode (see Supplementary Table S27).

#### Analysis 1—behavioral synchrony

To examine phase-related differences in behavioral synchrony, we fitted a linear mixed-effects model with phase of the FFSF paradigm (play vs. reunion) as a fixed effect (level 1), nested within dyads as a random intercept (level 2). This model allowed us to test whether behavioral synchrony systematically differed between the play and reunion phase of the FFSF paradigm, accounting for inter-dyad variability. The play phase served as the reference category in the intercept.

#### Analysis 2a—concurrent physiological synchrony

To analyze concurrent physiological synchrony across the phases of the FFSF paradigm, we computed a multilevel model to predict the cross-correlation of IBIs or RMSSD by the phase of the FFSF paradigm. The analysis was conducted with respect to concurrent synchrony, focusing exclusively on lag 0. We built a multilevel linear mixed-effects model with a two-level structure comprising three phases of the FFSF paradigm (fixed effect, level 1), nested in dyads (random effect, level 2) with phase 1 as reference (two dummy-coded variables). We included a random intercept effect (across participants), and due to model convergence problems, a fixed slope for phase. The model accounts for both within-dyad variability (fixed effect, phase) and between-dyad variability (random intercept). Mean-centered control variables included maternal and infant age as well as maternal BMI, known to influence HRV values. Separate models were computed for the cross-correlations of IBI and RMSSD. Estimated marginal means for each phase were tested against zero using Bonferroni correction to assess the presence of concurrent synchrony.

A sensitivity analysis was conducted using the *simr* package to estimate the minimum effect size that could be reliably detected (≥ 80% power) with the available sample size. Power was calculated for a range of hypothetical effect sizes (*β* = 0.05 to 0.30) for the predictor ‘phase’, based on the fitted model’s structure and variance components.

#### Analysis 2b—lagged physiological synchrony

We extended the model of Analysis 2a to a multilevel linear mixed-effects model with a three-level structure comprising the lags (-3 to 3; fixed effect, level 1), three phases of the FFSF paradigm (fixed effect, level 2), nested in dyads (random effect, level 3). Both factors, phase and lag, were treated as categorical and dummy-coded, with the play phase and lag 0 serving as the reference levels. To analyze phase-related differences in lagged cross-correlation, we included the interaction between lag and phase in the model. We included a random intercept effect (across participants) and control variables as described for Analysis 2a. Because differences between lags do not necessarily indicate whether synchrony at a given lag differs from zero, estimated marginal means were tested against zero using Bonferroni correction across lags within each phase. In cases of significant results, we re-ran the three-level model using cross-correlations from randomly paired dyads to test for pseudo-synchrony.

#### Analysis 3—biobehavioral synchrony

To assess the relationship between physiological and behavioral synchrony, we extended our three-level linear model. In this model, physiological cross-correlation (IBI or RMSSD) was predicted by lag (− 3 to 3; fixed effect, level 1), the three phases of the FFSF paradigm (fixed effect, level 2) and behavioral synchrony (fixed effect) with all effects nested within dyads (random effect, level 3). Given the relevance of lags, we included the interaction between behavioral synchrony and lag.

To reduce model complexity, analyses were conducted on cross-correlations for the play and reunion phases only. In these two-level models, phase-specific physiological cross-correlation (IBI or RMSSD) was predicted by lag (− 3 to 3; fixed effect, level 1) and phase-specific behavioral synchrony (fixed effect), nested within dyads (random effect, level 2).

Estimated marginal means were tested against zero for each of the seven lags, with Bonferroni correction, and separately for behavioral synchrony at the mean and *±* 1 SD.

#### Analysis 4—maternal bonding

The model used in Analysis 3 was extended to include maternal bonding as an additional fixed-effect predictor at level 3.

## Supplementary Information

Below is the link to the electronic supplementary material.

Supplementary Material 1

## Data Availability

The data that support the findings of this study are available from the corresponding author upon reasonable request.
