# Supplementary material for: Biobehavioral synchrony between three to four months old infants and psychologically healthy mothers during the face-to-face still-face paradigm
Source: Sci Rep. 2026 Aug 5;16:24136. doi: 10.1038/s41598-026-65050-1 (PMC13443841; doi:10.1038/s41598-026-65050-1)
Supplement: Supplementary file 1 [file 41598_2026_65050_MOESM1_ESM.docx]

**Biobehavioral Synchrony Between Three to Four Months Old Infants and Psychologically Healthy Mothers During the Face-to-Face Still-Face Paradigm**

**Supplement**

**Table S1**

*Results of the linear mixed model testing differences in behavioral synchrony between play and reunion phase of the FFSF paradigm (Analysis 1)*

| **Behavioral Synchrony** | | | | | | |
| --- | --- | --- | --- | --- | --- | --- |
| **Fixed effects** | | | | | | |
| Predictor | *b (SE)* | | *95%-CI* | | *p* | |
| Intercept | 3.71 (0.08) | | 3.55 – 3.88 | | **<.001** | |
| Phase (Reunion) | -0.29 (0.07) | | -0.42 – (-0.15) | | **<.001** | |
| **Random effects** | | | | | | |
| σ^2^ | | 0.20 | |  | |  |
| τ_00 id_ | | 0.39 | |  | |  |
| ICC | | 0.66 | |  | |  |
| N _id_ | | 87 | |  | |  |

*Note.* A linear mixed model (LMM) was fitted using REML with phase (Play vs. Reunion) as a fixed effect and a random intercept for each dyad to account for the paired structure.

Marginal R²: 0.034; Conditional R²: 0.668

**Table S2**

*Results of the linear mixed model examining phase differences in concurrent IBI synchrony across the FFSF paradigm (Analysis 2a)*

| **Concurrent IBI Synchrony** | | | | |  |
| --- | --- | --- | --- | --- | --- |
| **Fixed effects** | | | | |  |
| Predictor | *b (SE)* | | *95%-CI* | *p* | |
| Intercept | -0.06 (0.06) | | -0.18 – 0.05 | .275 | |
| Phase (Still-Face) | -0.03 (0.08) | | -0.19 – 0.13 | .750 | |
| Phase (Reunion) | -0.01 (0.08) | | -0.17 – 0.16 | .943 | |
| Maternal Age | -0.00 (0.01) | | -0.02 – 0.02 | .880 | |
| Infant Age | -0.18 (0.11) | | -0.39 – 0.04 | .105 | |
| Maternal BMI | 0.02 (0.01) | | -0.01 – 0.04 | .231 | |
| **Random effects** | | | | |  |
| σ^2^ | | 0.18 | | | |
| τ_00 id_ | | 0.00 | | | |
| ICC | | NA | | | |
| N _id_ | | 55 | | | |

*Note.* Multilevel linear mixed-effects model with a two-level structure comprising three phases of the FFSF paradigm (fixed effect, level 1), nested in dyads (random effect, level 2) with the play episode as reference. Singular fit, so conditional R² could not be estimated. Observations: 154; Marginal R²: 0.034

**Table S3**

*Results of the linear mixed model examining phase differences in concurrent RMSSD synchrony across the FFSF paradigm (Analysis 2a)*

| **Concurrent RMSSD Synchrony** | | | | |  |
| --- | --- | --- | --- | --- | --- |
| **Fixed effects** | | | | |  |
| Predictor | *b (SE)* | | *95%-CI* | *p* | |
| Intercept | -0.00 (0.05) | | -0.09 – 0.09 | .943 | |
| Phase (Still-Face) | -0.01 (0.06) | | -0.12 – 0.10 | .850 | |
| Phase (Reunion) | 0.02 (0.06) | | -0.10 – 0.14 | .738 | |
| Maternal Age | -0.00 (0.01) | | -0.02 – 0.02 | .810 | |
| Infant Age | 0.05 (0.10) | | -0.15 – 0.25 | .620 | |
| Maternal BMI | 0.00 (0.01) | | -0.02 – 0.02 | .994 | |
| **Random effects** | | | | |  |
| σ^2^ | | 0.08 | | | |
| τ_00 id_ | | 0.03 | | | |
| ICC | | 0.23 | | | |
| N _id_ | | 55 | | | |

*Note.* Multilevel linear mixed-effects model with a two-level structure comprising three phases of the FFSF paradigm (fixed effect, level 1), nested in dyads (random effect, level 2) with the play episode as reference. Observations: 154; Marginal R²: 0.004; Conditional R^2^: 0.236

**Table S4**

*Results of the linear mixed model testing effects of FFSF phase and lag on lagged IBI synchrony (Analysis 2b)*

| **Lagged IBI Synchrony** | | | | |  |
| --- | --- | --- | --- | --- | --- |
| **Fixed effects** | | | | |  |
| Predictor | *b (SE)* | | *95%-CI* | *p* | |
| Intercept | -0.07 (0.05) | | -0.16 – 0.02 | .153 | |
| Phase (Still-Face) | -0.03 (0.06) | | -0.15 – 0.10 | .686 | |
| Phase (Reunion) | -0.00 (0.06) | | -0.13 – 0.13 | .993 | |
| Lag -3 | 0.04 (0.06) | | -0.09 – 0.16 | .577 | |
| Lag -2 | 0.05 (0.06) | | -0.07 – 0.18 | .410 | |
| Lag -1 | 0.01 (0.06) | | -0.11 – 0.14 | .857 | |
| Lag 1 | 0.02 (0.06) | | -0.10 – 0.15 | .738 | |
| Lag 2 | 0.05 (0.06) | | -0.08 – 0.17 | .448 | |
| Lag 3 | 0.07 (0.06) | | -0.06 – 0.19 | .284 | |
| Maternal Age | -0.00 (0.00) | | -0.01 – 0.01 | .944 | |
| Infant Age | -0.08 (0.05) | | -0.17 – 0.01 | .091 | |
| Maternal BMI | 0.00 (0.01) | | -0.01 – 0.01 | .477 | |
| **Random effects** | | | | |  |
| σ^2^ | | 0.10 | | | |
| τ_00 id_ | | 0.01 | | | |
| ICC | | 0.07 | | | |
| N _id_ | | 55 | | | |

*Note.* Multilevel linear mixed-effects model with a three-level structure comprising the lags (-3 to 3; fixed effect, level 1), three phases of the FFSF paradigm (fixed effect, level 2), nested in dyads (random effect, level 3). 12 interaction terms (phase x lag) were not significant (all *p_s_* > .399)

Observations: 1078; Marginal R²: 0.022; Conditional R^2^: 0.086

**Table S5**

*Results of the linear mixed model testing effects of FFSF phase and lag on lagged RMSSD synchrony (Analysis 2b)*

| **Lagged RMSSD Synchrony** | | | | |  |
| --- | --- | --- | --- | --- | --- |
| **Fixed effects** | | | | |  |
| Predictor | *b (SE)* | | *95%-CI* | *p* | |
| Intercept | 0.00 (0.04) | | -0.08 – 0.08 | .982 | |
| Phase (Still-Face) | -0.01 (0.05) | | -0.12 – 0.09 | .787 | |
| Phase (Reunion) | 0.01 (0.06) | | -0.10 – 0.12 | .832 | |
| Lag -3 | 0.02 (0.06) | | -0.09 – 0.13 | .763 | |
| Lag -2 | 0.02 (0.06) | | -0.08 – 0.13 | .652 | |
| Lag -1 | -0.11 (0.06) | | -0.22 – (-0.00) | **.044** | |
| Lag 1 | -0.05 (0.06) | | -0.16 – 0.06 | .393 | |
| Lag 2 | -0.09 (0.06) | | -0.20 – 0.02 | .108 | |
| Lag 3 | 0.01 (0.06) | | -0.10 – 0.12 | .819 | |
| Maternal Age | 0.00 (0.00) | | -0.00 – 0.01 | .088 | |
| Infant Age | -0.04 (0.03) | | -0.10 – 0.02 | .235 | |
| Maternal BMI | 0.00 (0.00) | | -0.01 – 0.00 | .216 | |
| **Random effects** | | | | |  |
| σ^2^ | | 0.08 | | | |
| τ_00 id_ | | 0.00 | | | |
| ICC | | 0.02 | | | |
| N _id_ | | 55 | | | |

*Note.* Multilevel linear mixed-effects model with a three-level structure comprising the lags (-3 to 3; fixed effect, level 1), three phases of the FFSF paradigm (fixed effect, level 2), nested in dyads (random effect, level 3). 12 interaction terms (phase x lag) were not significant (all *p_s_* > .102)

Observations: 1078; Marginal R²: 0.025; Conditional R^2^: 0.041

**Table S6**

*Results of the linear mixed model examining lagged RMSSD synchrony as a function of FFSF phase and lag for surrogate pairs (Analysis 2b)*

| **Lagged RMSSD Synchrony** | | | | |  |
| --- | --- | --- | --- | --- | --- |
| **Fixed effects** | | | | |  |
| Predictor | *b (SE)* | | *95%-CI* | *p* | |
| Intercept | 0.06 (0.04) | | -0.01 – 0.13 | .100 | |
| Phase (Still-Face) | -0.07 (0.05) | | -0.17 – 0.03 | .158 | |
| Phase (Reunion) | -0.02 (0.05) | | -0.13 – 0.08 | .673 | |
| Lag -3 | -0.07 (0.05) | | -0.17 – 0.03 | .163 | |
| Lag -2 | -0.03 (0.05) | | -0.13 – 0.07 | .527 | |
| Lag -1 | -0.04 (0.05) | | -0.14 – 0.06 | .389 | |
| Lag 1 | -0.03 (0.05) | | -0.13 – 0.07 | .576 | |
| Lag 2 | -0.07 (0.05) | | -0.17 – 0.03 | .179 | |
| Lag 3 | -0.10 (0.05) | | -0.20 – 0.00 | .058 | |
| Maternal Age | 0.00 (0.00) | | -0.00 – 0.01 | .072 | |
| Infant Age | -0.03 (0.03) | | -0.08 – 0.02 | .256 | |
| Maternal BMI | 0.00 (0.00) | | -0.01 – 0.01 | .686 | |
| **Random effects** | | | | |  |
| σ^2^ | | 0.08 | | | |
| τ_00 id_ | | 0.00 | | | |
| ICC | | 0.01 | | | |
| N _id_ | | 54 | | | |

*Note.* Multilevel linear mixed-effects model with a three-level structure comprising the lags (-3 to 3; fixed effect, level 1), three phases of the FFSF paradigm (fixed effect, level 2), nested in pseudo-dyads (random effect, level 3). 12 interaction terms (phase x lag) were not significant (all *p_s_* > .086)

Observations: 1211; Marginal R²: 0.025; Conditional R^2^: 0.035

**Table S7**

*Results of the linear mixed model examining lagged RMSSD synchrony as a function of FFSF phase, lag, and behavioral synchrony (Analysis 3)*

| **Lagged RMSSD Synchrony** | | | |  |
| --- | --- | --- | --- | --- |
| **Fixed effects** | | | |  |
| Predictor | *b (SE)* | *95%-CI* | *p* | |
| Intercept | 0.19 (0.15) | -0.10 – 0.48 | .194 | |
| Behavioral Synchrony | -0.05 (0.04) | -0.13 – 0.03 | .193 | |
| Phase (Still-Face) | 0.04 (0.02) | -0.00 – 0.08 | .064 | |
| Phase (Reunion) | 0.01 (0.02) | -0.04 – 0.05 | .678 | |
| Lag -3 | -0.31 (0.20) | -0.71 – 0.10 | .135 | |
| Lag -2 | -0.22 (0.20) | -0.62 – 0.18 | .278 | |
| Lag -1 | -0.48 (0.20) | -0.88 – (-0.08) | **.019** | |
| Lag 1 | -0.25 (0.20) | -0.65 – 0.15 | .228 | |
| Lag 2 | -0.19 (0.20) | -0.59 – 0.21 | .350 | |
| Lag 3 | -0.17 (0.20) | -0.58 – 0.23 | .394 | |
| Behavioral Synchrony:lag-3 | 0.08 (0.06) | -0.03 – 0.19 | .136 | |
| Behavioral Synchrony:lag-2 | 0.06 (0.06) | -0.05 – 0.17 | .314 | |
| Behavioral Synchrony:lag-1 | 0.12 (0.06) | 0.01 – 0.23 | **.028** | |
| Behavioral Synchrony:lag 1 | 0.06 (0.06) | -0.05 – 0.17 | .256 | |
| Behavioral Synchrony:lag 2 | 0.04 (0.06) | -0.07 – 0.15 | .488 | |
| Behavioral Synchrony:lag 3 | 0.05 (0.06) | -0.07 – 0.16 | .420 | |
| Maternal Age | 0.00 (0.00) | 0.00 – 0.01 | .105 | |
| Infant Age | -0.03 (0.03) | -0.09 – 0.03 | .297 | |
| Maternal BMI | 0.00 (0.00) | -0.01 – 0.01 | .718 | |

*Table S7 continued on page 8*

*Table S7 continued*

| **Random effects** | |  |
| --- | --- | --- |
| σ^2^ | 0.08 | |
| τ_00 id_ | 0.00 | |
| ICC | 0.01 | |
| N _id_ | 48 | |

*Note.* Observations: 945; Marginal R²: 0.018; Conditional R^2^: 0.027

**Table S8**

*Results of the linear mixed model examining lagged IBI synchrony as a function of FFSF phase, lag, and behavioral synchrony (Analysis 3)*

| **Lagged IBI Synchrony** | | | | |  |
| --- | --- | --- | --- | --- | --- |
| **Fixed effects** | | | | |  |
| Predictor | *b (SE)* | | *95%-CI* | *p* | |
| Intercept | -0.16 (0.18) | | -0.51 – 0.19 | .366 | |
| Behavioral Synchrony | 0.02 (0.05) | | -0.08 – 0.12 | .661 | |
| Phase (Still-Face) | -0.01 (0.03) | | -0.06 – 0.04 | .801 | |
| Phase (Reunion) | 0.00 (0.03) | | -0.05 – 0.05 | .935 | |
| Lag -3 | 0.13 (0.23) | | -0.32 – 0.59 | .566 | |
| Lag -2 | 0.17 (0.23) | | -0.29 – 0.63 | .460 | |
| Lag -1 | 0.00 (0.23) | | -0.46 – 0.46 | 1.00 | |
| Lag 1 | -0.15 (0.23) | | -0.61 – 0.30 | .508 | |
| Lag 2 | 0.08 (0.23) | | -0.37 – 0.54 | .718 | |
| Lag 3 | 0.19 (0.23) | | -0.27 – 0.65 | .422 | |
| Maternal Age | 0.00 (0.00) | | -0.01 – 0.01 | .619 | |
| Infant Age | -0.06 (0.04) | | -0.15 – 0.03 | .168 | |
| Maternal BMI | 0.00 (0.01) | | -0.01 – 0.01 | .735 | |
| **Random effects** | | | | |  |
| σ^2^ | | 0.10 | | | |
| τ_00 id_ | | 0.01 | | | |
| ICC | | 0.05 | | | |
| N _id_ | | 48 | | | |

*Note.* Six interaction terms (Behavioral Synchrony x lag) were not significant (all *p_s_* > .412)

Observations: 945; Marginal R²: 0.016; Conditional R^2^: 0.063

**Table S9**

*Results of the linear mixed model testing effects of lag and behavioral synchrony on lagged RMSSD synchrony during the reunion phase (Analysis 3)*

| **Lagged RMSSD Synchrony (Reunion)** | | | |  |
| --- | --- | --- | --- | --- |
| **Fixed effects** | | | |  |
| Predictor | *b (SE)* | *95%-CI* | *p* | |
| Intercept | 0.02 (0.24) | -0.45 – 0.49 | .936 | |
| Behavioral Synchrony Reunion | -0.00 (0.07) | -0.13 – 0.13 | .982 | |
| Lag -3 | -0.15 (0.34) | -0.82 – 0.52 | .656 | |
| Lag -2 | -0.02 (0.34) | -0.69 – 0.65 | .954 | |
| Lag -1 | 0.08 (0.34) | -0.59 – 0.74 | .824 | |
| Lag 1 | 0.06 (0.34) | -0.61 – 0.72 | .870 | |
| Lag 2 | -0.12 (0.34) | -0.78 – 0.55 | .733 | |
| Lag 3 | -0.13 (0.34) | -0.80 – 0.54 | .701 | |
| Behavioral Synchrony:lag-3 | 0.04 (0.09) | -0.14 – 0.23 | .640 | |
| Behavioral Synchrony:lag-2 | -0.00 (0.09) | -0.19 – 0.18 | .977 | |
| Behavioral Synchrony:lag-1 | -0.01 (0.09) | -0.19 – 0.18 | .936 | |
| Behavioral Synchrony:lag 1 | -0.03 (0.09) | -0.22 – 0.16 | .755 | |
| Behavioral Synchrony:lag 2 | 0.02 (0.09) | -0.17 – 0.20 | .858 | |
| Behavioral Synchrony:lag 3 | 0.02 (0.09) | -0.17 – 0.20 | .864 | |
| Maternal Age | 0.01 (0.00) | -0.00 – 0.02 | .206 | |
| Infant Age | -0.03 (0.06) | -0.15 – 0.08 | .574 | |
| Maternal BMI | -0.00 (0.01) | -0.02 – 0.01 | .650 | |

*Table S9 continued on page 11*

*Table S9 continued*

| **Random effects** | |  |
| --- | --- | --- |
| σ^2^ | 0.09 | |
| τ_00 id_ | 0.00 | |
| ICC | NA | |
| N _id_ | 42 | |

*Note.* Random effect variance was negligible (singular fit), so conditional R² could not be estimated.

Observations: 294; Marginal R²: 0.026; Conditional R^2^: NA

**Table S10**

*Results of the linear mixed model testing effects of lag and behavioral synchrony on lagged RMSSD synchrony during the play phase (Analysis 3)*

| **Lagged RMSSD Synchrony (Play)** | | | |  |
| --- | --- | --- | --- | --- |
| **Fixed effects** | | | |  |
| Predictor | *b (SE)* | *95%-CI* | *p* | |
| Intercept | 0.38 (0.25) | -0.11 – 0.86 | .128 | |
| Behavioral Synchrony Play | -0.09 (0.07) | -0.22 – 0.04 | .173 | |
| Lag -3 | -0.40 (0.34) | -1.07 – 0.26 | .236 | |
| Lag -2 | -0.06 (0.34) | -0.73 – 0.60 | .851 | |
| Lag -1 | -0.99 (0.34) | -1.66 – (-0.32) | **.004** | |
| Lag 1 | -0.61 (0.34) | -1.27 – 0.06 | .075 | |
| Lag 2 | -0.44 (0.34) | -1.11 – 0.22 | .192 | |
| Lag 3 | -0.59 (0.34) | -1.26 – 0.08 | .082 | |
| Behavioral Synchrony:lag-3 | 0.10 (0.09) | -0.08 – 0.28 | .259 | |
| Behavioral Synchrony:lag-2 | 0.01 (0.09) | -0.17 – 0.19 | .886 | |
| Behavioral Synchrony:lag-1 | 0.22 (0.09) | 0.04 – 0.40 | **.016** | |
| Behavioral Synchrony:lag 1 | 0.15 (0.09) | -0.03 – 0.33 | .102 | |
| Behavioral Synchrony:lag 2 | 0.09 (0.09) | -0.09 – 0.27 | .338 | |
| Behavioral Synchrony:lag 3 | 0.15 (0.09) | -0.03 – 0.33 | .094 | |
| Maternal Age | 0.00 (0.00) | -0.01 – 0.01 | .976 | |
| Infant Age | -0.04 (0.05) | -0.14 – 0.06 | .426 | |
| Maternal BMI | -0.00 (0.01) | -0.02 – 0.01 | .617 | |

*Table S10 continued on page 13*

*Table S10 continued*

| **Random effects** | |  |
| --- | --- | --- |
| σ^2^ | 0.07 | |
| τ_00 id_ | 0.00 | |
| ICC | 0.03 | |
| N _id_ | 45 | |

*Note.* Observations: 315; Marginal R²: 0.077; Conditional R^2^: 0.102

**Table S11**

*Results of the linear mixed model examining associations between lagged IBI synchrony in the FFSF paradigm, behavioral synchrony, and maternal bonding (Analysis 4)*

| **Lagged IBI Synchrony** | | | |  |
| --- | --- | --- | --- | --- |
| **Fixed effects** | | | |  |
| Predictor | *b (SE)* | *95%-CI* | *p* | |
| Intercept | -0.22 (0.18) | -0.58 – 0.14 | .231 | |
| Behavioral Synchrony | 0.03 (0.05) | -0.07 – 0.12 | .587 | |
| Phase (Still-Face) | -0.01 (0.03) | -0.06 – 0.04 | .798 | |
| Phase (Reunion) | 0.00 (0.03) | -0.05 – 0.05 | .942 | |
| Lag -3 | 0.13 (0.23) | -0.32 – 0.59 | .566 | |
| Lag -2 | 0.17 (0.23) | -0.29 – 0.63 | .460 | |
| Lag -1 | 0.00 (0.23) | -0.46 – 0.46 | 1.000 | |
| Lag 1 | -0.15 (0.23) | -0.61 – 0.30 | .508 | |
| Lag 2 | 0.08 (0.23) | -0.37 – 0.54 | .718 | |
| Lag 3 | 0.19 (0.23) | -0.27 – 0.65 | .422 | |
| Behavioral Synchrony:lag-3 | -0.01 (0.06) | -0.14 – 0.11 | .824 | |
| Behavioral Synchrony:lag-2 | -0.03 (0.06) | -0.16 – 0.09 | .625 | |
| Behavioral Synchrony:lag-1 | 0.00 (0.06) | -0.12 – 0.13 | .946 | |
| Behavioral Synchrony:lag 1 | 0.05 (0.06) | -0.07 – 0.18 | .412 | |
| Behavioral Synchrony:lag 2 | -0.00 (0.06) | -0.13 – 0.12 | .966 | |
| Behavioral Synchrony:lag 3 | -0.03 (0.06) | -0.16 – 0.09 | .606 | |
| PBQ-16 | 0.01 (0.00) | -0.00 – 0.01 | .118 | |
| Maternal Age | 0.00 (0.00) | -0.01 – 0.01 | .590 | |
| Infant Age | -0.06 (0.04) | -0.14 – 0.03 | .202 | |
| Maternal BMI | -0.00 (0.01) | -0.01 – 0.01 | .799 | |

*Table S11 continued on page 15*

*Table S11 continued*

| **Random effects** | |  |
| --- | --- | --- |
| σ^2^ | 0.10 | |
| τ_00 id_ | 0.00 | |
| ICC | 0.05 | |
| N _id_ | 48 | |

*Note.* Observations: 945; Marginal R²: 0.021; Conditional R^2^: 0.065; PBQ-16 = 16-item Postpartum Bonding Questionnaire

**Table S12**

*Results of the linear mixed model examining associations between lagged RMSSD synchrony in the FFSF paradigm, behavioral synchrony, and maternal bonding (Analysis 4)*

| **Lagged RMSSD Synchrony** | | | |  |
| --- | --- | --- | --- | --- |
| **Fixed effects** | | | |  |
| Predictor | *b (SE)* | *95%-CI* | *p* | |
| Intercept | 0.21 (0.15) | -0.09 – 0.50 | .173 | |
| Behavioral Synchrony | -0.05 (0.04) | -0.13 – 0.03 | .185 | |
| Phase (Still-Face) | 0.04 (0.02) | -0.00 – 0.08 | .064 | |
| Phase (Reunion) | 0.01 (0.02) | -0.04 – 0.05 | .671 | |
| Lag -3 | -0.31 (0.20) | -0.71 – 0.10 | .135 | |
| Lag -2 | -0.22 (0.20) | -0.62 – 0.18 | .277 | |
| Lag -1 | -0.48 (0.20) | -0.88 – (-0.08) | **.019** | |
| Lag 1 | -0.25 (0.20) | -0.65 – 0.15 | .228 | |
| Lag 2 | -0.19 (0.20) | -0.59 – 0.21 | .349 | |
| Lag 3 | -0.17 (0.20) | -0.58 – 0.23 | .394 | |
| Behavioral Synchrony:lag-3 | 0.08 (0.06) | -0.03 – 0.19 | .136 | |
| Behavioral Synchrony:lag-2 | 0.06 (0.06) | -0.05 – 0.17 | .314 | |
| Behavioral Synchrony:lag-1 | 0.12 (0.06) | 0.01 – 0.23 | **.028** | |
| Behavioral Synchrony:lag 1 | 0.06 (0.06) | -0.05 – 0.17 | .256 | |
| Behavioral Synchrony:lag 2 | 0.04 (0.06) | -0.07 – 0.15 | .488 | |
| Behavioral Synchrony:lag 3 | 0.05 (0.06) | -0.07 – 0.16 | .420 | |
| PBQ-16 | -0.00 (0.00) | -0.01 – 0.00 | .620 | |
| Maternal Age | 0.00 (0.00) | -0.00 – 0.01 | .112 | |
| Infant Age | -0.03 (0.03) | -0.09 – 0.03 | .287 | |
| Maternal BMI | 0.00 (0.00) | -0.01 – 0.01 | .736 | |

*Table S12 continued on page 17*

*Table S12 continued*

| **Random effects** | |  |
| --- | --- | --- |
| σ^2^ | 0.08 | |
| τ_00 id_ | 0.00 | |
| ICC | 0.01 | |
| N _id_ | 48 | |

*Note.* Observations: 945; Marginal R²: 0.018; Conditional R^2^: 0.028; PBQ-16 = 16-item Postpartum Bonding Questionnaire

**Table S13**

*Correlations of relevant study variables with 95% confidence intervals*

| Variable | 1 | 2 | 3 | 4 | 5 | 6 | 7 | 8 | 9 |
| --- | --- | --- | --- | --- | --- | --- | --- | --- | --- |
|  |  |  |  |  |  |  |  |  |  |
| 1. Cross-correlations logRMSSD |  |  |  |  |  |  |  |  |  |
|  |  |  |  |  |  |  |  |  |  |
| 2. Cross-correlations IBI | **.16**** |  |  |  |  |  |  |  |  |
|  | [.10, .22] |  |  |  |  |  |  |  |  |
|  |  |  |  |  |  |  |  |  |  |
| 3. Maternal age | .05 | -.03 |  |  |  |  |  |  |  |
|  | [-.01, .11] | [-.09, .03] |  |  |  |  |  |  |  |
|  |  |  |  |  |  |  |  |  |  |
| 4. Infant age (months) | -.02 | **-.08**** | **.27**** |  |  |  |  |  |  |
|  | [-.08, .04] | [-.14, -.02] | [.21, .32] |  |  |  |  |  |  |
|  |  |  |  |  |  |  |  |  |  |
| 5. Maternal BMI | -.04 | .05 | .00 | **-.13**** |  |  |  |  |  |
|  | [-.10, .02] | [-.01, .11] | [-.06, .06] | [-.19, -.07] |  |  |  |  |  |
|  |  |  |  |  |  |  |  |  |  |
| 6. Infant size (birth) | -.04 | .01 | **-.13**** | **-.08*** | **-.09**** |  |  |  |  |
|  | [-.09, .02] | [-.05, .07] | [-.19, -.07] | [-.13, -.02] | [-.15, -.03] |  |  |  |  |
|  |  |  |  |  |  |  |  |  |  |
| 7. Infant weight (birth) | -.04 | .05 | -.01 | **.07*** | **-.11**** | **.67**** |  |  |  |
|  | [-.09, .02] | [-.01, .11] | [-.07, .04] | [.01, .13] | [-.17, -.05] | [.63, .70] |  |  |  |
|  |  |  |  |  |  |  |  |  |  |
| 8. EPDS | -.05 | **.08*** | -.04 | .04 | **-.12**** | **-.13**** | -.00 |  |  |
|  | [-.11, .01] | [.02, .13] | [-.10, .02] | [-.02, .10] | [-.18, -.06] | [-.18, -.07] | [-.06, .06] |  |  |
|  |  |  |  |  |  |  |  |  |  |
| 9. PBQ-16 | -.02 | .06 | .01 | -.01 | -.05 | -.05 | **.12**** | **.51**** |  |
|  | [-.08, .04] | [-.00, .12] | [-.05, .07] | [-.07, .05] | [-.11, .01] | [-.10, .01] | [.06, .18] | [.47, .55] |  |
|  |  |  |  |  |  |  |  |  |  |
| 10. Behavioral Synchrony Score | .01 | .04 | **-.16**** | **-.18**** | **.19**** | **.17**** | **.12**** | **-.32**** | **-.14**** |
|  | [-.05, .08] | [-.02, .10] | [-.22, -.10] | [-.24, -.12] | [.13, .25] | [.11, .23] | [.06, .19] | [-.38, -.26] | [-.20, -.08] |

*Note.* Pearson correlation coefficients. Only participants with complete data on all variables were included in the analyses. Values in square brackets indicate the 95% confidence interval for each correlation. * indicates *p* < .05. ** indicates *p* < .01.

**Table S14**

*Results of the linear mixed model examining phase differences in concurrent IBI synchrony across the FFSF paradigm, based on Fisher z-transformed cross-correlation coefficients (Analysis 2a)*

| **Concurrent IBI Synchrony** | | | | |  |
| --- | --- | --- | --- | --- | --- |
| **Fixed effects** | | | | |  |
| Predictor | *b (SE)* | | *95%-CI* | *p* | |
| Intercept | -0.05 (0.07) | | -0.20 – 0.09 | .468 | |
| Phase (Still-Face) | -0.06 (0.10) | | -0.26 – 0.14 | .523 | |
| Phase (Reunion) | -0.04 (0.10) | | -0.25 – 0.16 | .682 | |
| Maternal Age | -0.00 (0.01) | | -0.03 – 0.02 | .834 | |
| Infant Age | -0.22 (0.13) | | -0.49 – 0.04 | .098 | |
| Maternal BMI | 0.02 (0.02) | | -0.01 – 0.05 | .275 | |
| **Random effects** | | | | |  |
| σ^2^ | | 0.27 | | | |
| τ_00 id_ | | 0.00 | | | |
| ICC | | NA | | | |
| N _id_ | | 55 | | | |

*Note.* Multilevel linear mixed-effects model with a two-level structure comprising three phases of the FFSF paradigm (fixed effect, level 1), nested in dyads (random effect, level 2) with the play episode as reference. Singular fit, so conditional R² could not be estimated. Observations: 154; Marginal R²: 0.035

**Table S15**

*Results of the linear mixed model examining phase differences in concurrent RMSSD synchrony across the FFSF paradigm, based on Fisher z-transformed cross-correlation coefficients (Analysis 2a)*

| **Concurrent RMSSD Synchrony** | | | | |  |
| --- | --- | --- | --- | --- | --- |
| **Fixed effects** | | | | |  |
| Predictor | *b (SE)* | | *95%-CI* | *p* | |
| Intercept | 0.00 (0.05) | | -0.10 – 0.10 | .995 | |
| Phase (Still-Face) | -0.01 (0.06) | | -0.14 – 0.11 | .869 | |
| Phase (Reunion) | 0.01 (0.07) | | -0.12 – 0.14 | .841 | |
| Maternal Age | -0.00 (0.01) | | -0.02 – 0.02 | .885 | |
| Infant Age | 0.05 (0.11) | | -0.17 – 0.27 | .642 | |
| Maternal BMI | -0.00 (0.01) | | -0.03 – 0.02 | .952 | |
| **Random effects** | | | | |  |
| σ^2^ | | 0.10 | | | |
| τ_00 id_ | | 0.03 | | | |
| ICC | | 0.23 | | | |
| N _id_ | | 55 | | | |

*Note.* Multilevel linear mixed-effects model with a two-level structure comprising three phases of the FFSF paradigm (fixed effect, level 1), nested in dyads (random effect, level 2) with the play episode as reference. Observations: 154; Marginal R²: 0.003; Conditional R^2^: 0.233

**Table S16**

*Results of the linear mixed model testing effects of FFSF phase and lag on lagged IBI synchrony, based on Fisher z-transformed cross-correlation coefficients (Analysis 2b)*

| **Lagged IBI Synchrony** | | | | |  |
| --- | --- | --- | --- | --- | --- |
| **Fixed effects** | | | | |  |
| Predictor | *b (SE)* | | *95%-CI* | *p* | |
| Intercept | -0.06 (0.05) | | -0.16 – 0.05 | .291 | |
| Phase (Still-Face) | -0.06 (0.07) | | -0.20 – 0.08 | .368 | |
| Phase (Reunion) | -0.04 (0.07) | | -0.18 – 0.11 | .630 | |
| Lag -3 | 0.03 (0.07) | | -0.12 – 0.17 | .725 | |
| Lag -2 | 0.04 (0.07) | | -0.10 – 0.18 | .555 | |
| Lag -1 | -0.01 (0.07) | | -0.15 – 0.13 | .875 | |
| Lag 1 | 0.00 (0.07) | | -0.14 – 0.15 | .948 | |
| Lag 2 | 0.04 (0.07) | | -0.11 – 0.18 | .624 | |
| Lag 3 | 0.06 (0.07) | | -0.08 – 0.20 | .418 | |
| Maternal Age | -0.00 (0.00) | | -0.01 – 0.01 | .966 | |
| Infant Age | -0.09 (0.05) | | -0.20 – 0.01 | .086 | |
| Maternal BMI | 0.00 (0.01) | | -0.01 – 0.02 | .496 | |
| **Random effects** | | | | |  |
| σ^2^ | | 0.13 | | | |
| τ_00 id_ | | 0.01 | | | |
| ICC | | 0.07 | | | |
| N _id_ | | 55 | | | |

*Note.* Multilevel linear mixed-effects model with a three-level structure comprising the lags (-3 to 3; fixed effect, level 1), three phases of the FFSF paradigm (fixed effect, level 2), nested in dyads (random effect, level 3). 12 interaction terms (phase x lag) were not significant (all *p_s_* > .274)

Observations: 1078; Marginal R²: 0.023; Conditional R^2^: 0.090

**Table S17**

*Results of the linear mixed model testing effects of FFSF phase and lag on lagged RMSSD synchrony, based on Fisher z-transformed cross-correlation coefficients (Analysis 2b)*

| **Lagged RMSSD Synchrony** | | | | |  |
| --- | --- | --- | --- | --- | --- |
| **Fixed effects** | | | | |  |
| Predictor | *b (SE)* | | *95%-CI* | *p* | |
| Intercept | 0.00 (0.04) | | -0.08 – 0.09 | .912 | |
| Phase (Still-Face) | -0.01 (0.06) | | -0.13 – 0.10 | .808 | |
| Phase (Reunion) | 0.00 (0.06) | | -0.12 – 0.13 | .940 | |
| Lag -3 | 0.01 (0.06) | | -0.10 – 0.13 | .807 | |
| Lag -2 | 0.02 (0.06) | | -0.10 – 0.14 | .704 | |
| Lag -1 | -0.12 (0.06) | | -0.24 – (-0.00) | **.042** | |
| Lag 1 | -0.05 (0.06) | | -0.17 – 0.06 | .367 | |
| Lag 2 | -0.10 (0.06) | | -0.22 – 0.02 | .108 | |
| Lag 3 | 0.01 (0.06) | | -0.11 – 0.13 | .867 | |
| Maternal Age | 0.01 (0.00) | | -0.00 – 0.01 | .082 | |
| Infant Age | -0.04 (0.03) | | -0.11 – 0.03 | .241 | |
| Maternal BMI | -0.01 (0.00) | | -0.01 – 0.00 | .187 | |
| **Random effects** | | | | |  |
| σ^2^ | | 0.09 | | | |
| τ_00 id_ | | 0.00 | | | |
| ICC | | 0.02 | | | |
| N _id_ | | 55 | | | |

*Note.* Multilevel linear mixed-effects model with a three-level structure comprising the lags (-3 to 3; fixed effect, level 1), three phases of the FFSF paradigm (fixed effect, level 2), nested in dyads (random effect, level 3). 12 interaction terms (phase x lag) were not significant (all *p_s_* > .064)

Observations: 1078; Marginal R²: 0.025; Conditional R^2^: 0.041

**Table S18**

*Results of the linear mixed model examining lagged RMSSD synchrony as a function of FFSF phase and lag for surrogate pairs, based on Fisher z-transformed cross-correlation coefficients (Analysis 2b)*

| **Lagged RMSSD Synchrony** | | | | |  |
| --- | --- | --- | --- | --- | --- |
| **Fixed effects** | | | | |  |
| Predictor | *b (SE)* | | *95%-CI* | *p* | |
| Intercept | 0.07 (0.04) | | -0.01 – 0.15 | .088 | |
| Phase (Still-Face) | -0.09 (0.06) | | -0.19 – 0.02 | .123 | |
| Phase (Reunion) | -0.03 (0.06) | | -0.14 – 0.09 | .648 | |
| Lag -3 | -0.08 (0.06) | | -0.19 – 0.03 | .138 | |
| Lag -2 | -0.04 (0.06) | | -0.15 – 0.07 | .484 | |
| Lag -1 | -0.05 (0.06) | | -0.16 – 0.06 | .385 | |
| Lag 1 | -0.03 (0.06) | | -0.14 – 0.07 | .532 | |
| Lag 2 | -0.08 (0.06) | | -0.19 – 0.03 | .146 | |
| Lag 3 | -0.11 (0.06) | | -0.22 – 0.00 | .052 | |
| Maternal Age | 0.01 (0.00) | | -0.00 – 0.01 | .070 | |
| Infant Age | -0.03 (0.03) | | -0.09 – 0.03 | .269 | |
| Maternal BMI | 0.00 (0.00) | | -0.01 – 0.01 | .688 | |
| **Random effects** | | | | |  |
| σ^2^ | | 0.09 | | | |
| τ_00 id_ | | 0.00 | | | |
| ICC | | 0.01 | | | |
| N _id_ | | 54 | | | |

*Note.* Multilevel linear mixed-effects model with a three-level structure comprising the lags (-3 to 3; fixed effect, level 1), three phases of the FFSF paradigm (fixed effect, level 2), nested in pseudo-dyads (random effect, level 3). 12 interaction terms (phase x lag) were not significant (all *p_s_* > .085)

Observations: 1211; Marginal R²: 0.026; Conditional R^2^: 0.037

**Table S19**

*Results of the linear mixed model examining lagged RMSSD synchrony as a function of FFSF phase, lag, and behavioral synchrony, based on Fisher z-transformed cross-correlation coefficients (Analysis 3)*

| **Lagged RMSSD Synchrony** | | | |  |
| --- | --- | --- | --- | --- |
| **Fixed effects** | | | |  |
| Predictor | *b (SE)* | *95%-CI* | *p* | |
| Intercept | 0.23 (0.16) | -0.09 – 0.55 | .155 | |
| Behavioral Synchrony | -0.06 (0.04) | -0.15 – 0.02 | .160 | |
| Phase (Still-Face) | 0.04 (0.02) | -0.00 – 0.09 | .072 | |
| Phase (Reunion) | 0.01 (0.02) | -0.04 – 0.06 | .704 | |
| Lag -3 | -0.35 (0.22) | -0.79 – 0.09 | .120 | |
| Lag -2 | -0.25 (0.22) | -0.69 – 0.19 | .273 | |
| Lag -1 | -0.54 (0.22) | -0.98 – (-0.10) | **.017** | |
| Lag 1 | -0.31 (0.22) | -0.75 – 0.13 | .168 | |
| Lag 2 | -0.24 (0.22) | -0.68 – 0.20 | .281 | |
| Lag 3 | -0.21 (0.22) | -0.65 – 0.23 | .343 | |
| Behavioral Synchrony:lag-3 | 0.10 (0.06) | -0.03 – 0.22 | .124 | |
| Behavioral Synchrony:lag-2 | 0.06 (0.06) | -0.06 – 0.18 | .310 | |
| Behavioral Synchrony:lag-1 | 0.14 (0.06) | 0.02 – 0.26 | **.024** | |
| Behavioral Synchrony:lag 1 | 0.08 (0.06) | -0.04 – 0.20 | .197 | |
| Behavioral Synchrony:lag 2 | 0.05 (0.06) | -0.07 – 0.17 | .399 | |
| Behavioral Synchrony:lag 3 | 0.05 (0.06) | -0.07 – 0.18 | .377 | |
| Maternal Age | 0.00 (0.00) | -0.00 – 0.01 | .106 | |
| Infant Age | -0.04 (0.03) | -0.10 – 0.03 | .290 | |
| Maternal BMI | 0.00 (0.00) | -0.01 – 0.01 | .742 | |

*Table S19 continued*

| **Random effects** | |  |
| --- | --- | --- |
| σ^2^ | 0.09 | |
| τ_00 id_ | 0.00 | |
| ICC | 0.01 | |
| N _id_ | 48 | |

*Note.* Observations: 945; Marginal R²: 0.017; Conditional R^2^: 0.027

**Table S20**

*Results of the linear mixed model examining lagged IBI synchrony as a function of FFSF phase, lag, and behavioral synchrony, based on Fisher z-transformed cross-correlation coefficients (Analysis 3)*

| **Lagged IBI Synchrony** | | | | |  |
| --- | --- | --- | --- | --- | --- |
| **Fixed effects** | | | | |  |
| Predictor | *b (SE)* | | *95%-CI* | *p* | |
| Intercept | -0.16 (0.21) | | -0.56 – 0.24 | .438 | |
| Behavioral Synchrony | 0.02 (0.06) | | -0.09 – 0.13 | .755 | |
| Phase (Still-Face) | -0.01 (0.03) | | -0.07 – 0.04 | .621 | |
| Phase (Reunion) | -0.00 (0.03) | | -0.06 – 0.05 | .885 | |
| Lag -3 | 0.14 (0.27) | | -0.38 – 0.67 | .588 | |
| Lag -2 | 0.18 (0.27) | | -0.35 – 0.70 | .510 | |
| Lag -1 | -0.03 (0.27) | | -0.55 – 0.49 | .914 | |
| Lag 1 | -0.17 (0.27) | | -0.69 – 0.36 | .531 | |
| Lag 2 | 0.09 (0.27) | | -0.44 – 0.61 | .746 | |
| Lag 3 | 0.18 (0.27) | | -0.34 – 0.70 | .491 | |
| Maternal Age | 0.00 (0.00) | | -0.01 – 0.01 | .617 | |
| Infant Age | -0.07 (0.05) | | -0.18 – 0.03 | .152 | |
| Maternal BMI | -0.00 (0.01) | | -0.02 – 0.01 | .774 | |
| **Random effects** | | | | |  |
| σ^2^ | | 0.13 | | | |
| τ_00 id_ | | 0.01 | | | |
| ICC | | 0.05 | | | |
| N _id_ | | 48 | | | |

*Note.* Six interaction terms (Behavioral Synchrony x lag) were not significant (all *p_s_* > .428)

Observations: 945; Marginal R²: 0.016; Conditional R^2^: 0.068

**Table S21**

*Results of the linear mixed model testing effects of lag and behavioral synchrony on lagged RMSSD synchrony during the reunion phase, based on Fisher z-transformed cross-correlation coefficients (Analysis 3)*

| **Lagged RMSSD Synchrony (Reunion)** | | | |  |
| --- | --- | --- | --- | --- |
| **Fixed effects** | | | |  |
| Predictor | *b (SE)* | *95%-CI* | *p* | |
| Intercept | 0.02 (0.27) | -0.51 – 0.54 | .945 | |
| Behavioral Synchrony Reunion | -0.00 (0.07) | -0.15 – 0.15 | .981 | |
| Lag -3 | -0.15 (0.38) | -0.89 – 0.59 | .690 | |
| Lag -2 | 0.00 (0.38) | -0.74 – 0.74 | .994 | |
| Lag -1 | 0.13 (0.38) | -0.61 – 0.87 | .725 | |
| Lag 1 | 0.06 (0.38) | -0.68 – 0.80 | .870 | |
| Lag 2 | -0.11 (0.38) | -0.85 – 0.63 | .761 | |
| Lag 3 | -0.15 (0.38) | -0.89 – 0.59 | .698 | |
| Behavioral Synchrony:lag-3 | 0.04 (0.10) | -0.16 – 0.25 | .669 | |
| Behavioral Synchrony:lag-2 | -0.01 (0.10) | -0.21 – 0.20 | .946 | |
| Behavioral Synchrony:lag-1 | -0.02 (0.10) | -0.23 – 0.19 | .853 | |
| Behavioral Synchrony:lag 1 | -0.03 (0.10) | -0.24 – 0.18 | .765 | |
| Behavioral Synchrony:lag 2 | 0.02 (0.10) | -0.19 – 0.22 | .872 | |
| Behavioral Synchrony:lag 3 | 0.02 (0.10) | -0.19 – 0.23 | .855 | |
| Maternal Age | 0.01 (0.01) | -0.00 – 0.02 | .181 | |
| Infant Age | -0.03 (0.07) | -0.16 – 0.10 | .637 | |
| Maternal BMI | -0.00 (0.01) | -0.02 – 0.01 | .672 | |

*Table S21 continued on page 28*

*Table S21 continued*

| **Random effects** | |  |
| --- | --- | --- |
| σ^2^ | 0.10 | |
| τ_00 id_ | 0.00 | |
| ICC | NA | |
| N _id_ | 42 | |

*Note.* Random effect variance was negligible (singular fit), so conditional R² could not be estimated.

Observations: 294; Marginal R²: 0.027; Conditional R^2^: NA

**Table S22**

*Results of the linear mixed model testing effects of lag and behavioral synchrony on lagged RMSSD synchrony during the play phase, based on Fisher z-transformed cross-correlation coefficients (Analysis 3)*

| **Lagged RMSSD Synchrony (Play)** | | | |  |
| --- | --- | --- | --- | --- |
| **Fixed effects** | | | |  |
| Predictor | *b (SE)* | *95%-CI* | *p* | |
| Intercept | 0.45 (0.27) | -0.08 – 0.97 | .096 | |
| Behavioral Synchrony Play | -0.11 (0.07) | -0.25 – 0.03 | .139 | |
| Lag -3 | -0.48 (0.37) | -1.20 – 0.24 | .193 | |
| Lag -2 | -0.10 (0.37) | -0.82 – 0.62 | .787 | |
| Lag -1 | -1.14 (0.37) | -1.86 – (-0.41) | **.002** | |
| Lag 1 | -0.71 (0.37) | -1.44 – 0.01 | .054 | |
| Lag 2 | -0.51 (0.37) | -1.24 – 0.21 | .165 | |
| Lag 3 | -0.69 (0.37) | -1.41 – 0.03 | .062 | |
| Behavioral Synchrony:lag-3 | 0.12 (0.10) | -0.07 – 0.32 | .220 | |
| Behavioral Synchrony:lag-2 | 0.02 (0.10) | -0.17 – 0.22 | .836 | |
| Behavioral Synchrony:lag-1 | 0.26 (0.10) | 0.06 – 0.45 | **.010** | |
| Behavioral Synchrony:lag 1 | 0.17 (0.10) | -0.02 – 0.37 | .078 | |
| Behavioral Synchrony:lag 2 | 0.10 (0.10) | -0.09 – 0.30 | .305 | |
| Behavioral Synchrony:lag 3 | 0.18 (0.10) | -0.02 – 0.37 | .074 | |
| Maternal Age | -0.00 (0.00) | -0.01 – 0.01 | .966 | |
| Infant Age | -0.04 (0.05) | -0.15 – 0.06 | .448 | |
| Maternal BMI | -0.00 (0.01) | -0.02 – 0.01 | .611 | |

*Table S22 continued on page 30*

*Table S22 continued*

| **Random effects** | |  |
| --- | --- | --- |
| σ^2^ | 0.09 | |
| τ_00 id_ | 0.00 | |
| ICC | 0.03 | |
| N _id_ | 45 | |

*Note.* Observations: 315; Marginal R²: 0.080; Conditional R^2^: 0.104

**Table S23**

*Results of the linear mixed model examining associations between lagged IBI synchrony in the FFSF paradigm, behavioral synchrony, and maternal bonding, based on Fisher z-transformed cross-correlation coefficients (Analysis 4)*

| **Lagged IBI Synchrony** | | | |  |
| --- | --- | --- | --- | --- |
| **Fixed effects** | | | |  |
| Predictor | *b (SE)* | *95%-CI* | *p* | |
| Intercept | -0.23 (0.21) | -0.64 – 0.18 | .277 | |
| Behavioral Synchrony | 0.02 (0.06) | -0.09 – 0.13 | .673 | |
| Phase (Still-Face) | -0.01 (0.03) | -0.07 – 0.04 | .618 | |
| Phase (Reunion) | -0.00 (0.03) | -0.06 – 0.05 | .878 | |
| Lag -3 | 0.14 (0.27) | -0.38 – 0.67 | .588 | |
| Lag -2 | 0.18 (0.27) | -0.35 – 0.70 | .510 | |
| Lag -1 | -0.03 (0.27) | -0.55 – 0.49 | .914 | |
| Lag 1 | -0.17 (0.27) | -0.69 – 0.36 | .531 | |
| Lag 2 | 0.09 (0.27) | -0.44 – 0.61 | .746 | |
| Lag 3 | 0.18 (0.27) | -0.34 – 0.70 | .491 | |
| Behavioral Synchrony:lag-3 | -0.01 (0.07) | -0.16 – 0.13 | .864 | |
| Behavioral Synchrony:lag-2 | -0.03 (0.07) | -0.17 – 0.12 | .704 | |
| Behavioral Synchrony:lag-1 | 0.01 (0.07) | -0.13 – 0.16 | .845 | |
| Behavioral Synchrony:lag 1 | 0.06 (0.07) | -0.09 – 0.20 | .428 | |
| Behavioral Synchrony:lag 2 | 0.00 (0.07) | -0.14 – 0.14 | .988 | |
| Behavioral Synchrony:lag 3 | -0.03 (0.07) | -0.17 – 0.12 | .701 | |
| PBQ-16 | 0.01 (0.00) | -0.00 – 0.01 | .109 | |
| Maternal Age | 0.00 (0.00) | -0.01 – 0.01 | .588 | |
| Infant Age | -0.07 (0.05) | -0.17 – 0.03 | .183 | |
| Maternal BMI | -0.00 (0.01) | -0.01 – 0.01 | .841 | |

*Table S23 continued on page 32*

*Table S23 continued*

| **Random effects** | |  |
| --- | --- | --- |
| σ^2^ | 0.13 | |
| τ_00 id_ | 0.01 | |
| ICC | 0.05 | |
| N _id_ | 48 | |

*Note.* Observations: 945; Marginal R²: 0.022; Conditional R^2^: 0.070; PBQ-16 = 16-item Postpartum Bonding Questionnaire

**Table S24**

*Results of the linear mixed model examining associations between lagged RMSSD synchrony in the FFSF paradigm, behavioral synchrony, and maternal bonding, based on Fisher z-transformed cross-correlation coefficients (Analysis 4)*

| **Lagged RMSSD Synchrony** | | | |  |
| --- | --- | --- | --- | --- |
| **Fixed effects** | | | |  |
| Predictor | *b (SE)* | *95%-CI* | *p* | |
| Intercept | 0.24 (0.17) | -0.08 – 0.57 | .143 | |
| Behavioral Synchrony | -0.06 (0.04) | -0.15 – 0.02 | .155 | |
| Phase (Still-Face) | 0.04 (0.02) | -0.00 – 0.09 | .072 | |
| Phase (Reunion) | 0.01 (0.02) | -0.04 – 0.06 | .698 | |
| Lag -3 | -0.35 (0.22) | -0.79 – 0.09 | .120 | |
| Lag -2 | -0.25 (0.22) | -0.69 – 0.19 | .273 | |
| Lag -1 | -0.54 (0.22) | -0.98 – (-0.10) | **.017** | |
| Lag 1 | -0.31 (0.22) | -0.75 – 0.13 | .168 | |
| Lag 2 | -0.24 (0.22) | -0.68 – 0.20 | .281 | |
| Lag 3 | -0.21 (0.22) | -0.65 – 0.23 | .343 | |
| Behavioral Synchrony:lag-3 | 0.10 (0.06) | -0.03 – 0.22 | .124 | |
| Behavioral Synchrony:lag-2 | 0.06 (0.06) | -0.06 – 0.18 | .310 | |
| Behavioral Synchrony:lag-1 | 0.14 (0.06) | 0.02 – 0.26 | **.024** | |
| Behavioral Synchrony:lag 1 | 0.08 (0.06) | -0.04 – 0.20 | .197 | |
| Behavioral Synchrony:lag 2 | 0.05 (0.06) | -0.07 – 0.17 | .399 | |
| Behavioral Synchrony:lag 3 | 0.05 (0.06) | -0.07 – 0.18 | .377 | |
| PBQ-16 | -0.00 (0.00) | -0.01 – 0.00 | .700 | |
| Maternal Age | 0.00 (0.00) | -0.00 – 0.01 | .112 | |
| Infant Age | -0.04 (0.03) | -0.10 – 0.03 | .285 | |
| Maternal BMI | 0.00 (0.00) | -0.01 – 0.01 | .756 | |

*Table S24 continued on page 34*

*Table S24 continued*

| **Random effects** | |  |
| --- | --- | --- |
| σ^2^ | 0.09 | |
| τ_00 id_ | 0.00 | |
| ICC | 0.01 | |
| N _id_ | 48 | |

*Note.* Observations: 945; Marginal R²: 0.017; Conditional R^2^: 0.028; PBQ-16 = 16-item Postpartum Bonding Questionnaire

**Table S25**

*Descriptive statistics for behavioral code infant negative affect*

| **Infant negative affect** | | | |
| --- | --- | --- | --- |
| Phase of the FFSF paradigm | *n* | *M* | *SD* |
| Play | 87 | 1.45 | 0.81 |
| Reunion | 87 | 2.08 | 1.32 |

*Note.* Negative affect was operationalized using the Coding Interactive Behavior (CIB) *Negative Emotionality* code, capturing verbal and nonverbal expressions of negative emotions.

**Table S26**

*Results of linear mixed model testing differences in infant negative affect between play and reunion phase of the FFSF paradigm*

| **Infant negative affect** | | | | | | | |  |
| --- | --- | --- | --- | --- | --- | --- | --- | --- |
| **Fixed effects** | | | | | | | |  |
| Predictor | *Estimates* | | *95%-CI* | | *p* | | *ß* | |
| Intercept | 1.45 | | 1.22 – 1.68 | | **<.001** | |  | |
| Phase (Reunion) | 0.63 | | 0.42 – 0.84 | | **<.001** | | .56 | |
| **Random effects** | | | | | | | |  |
| σ^2^ | | 0.50 | |  | |  | |  |
| τ_00 id_ | | 0.70 | |  | |  | |  |
| ICC | | 0.58 | |  | |  | |  |
| N _id_ | | 87 | |  | |  | |  |

*Note.* A linear mixed model (LMM) was fitted using REML with phase (Play vs. Reunion) as a fixed effect and a random intercept for each infant to account for the paired structure.

Marginal R² / Conditional R²: 0.077 / 0.617

**Table S27**

*Demographic characteristics by availability of physiological synchrony data*

|  | Dyads without physiological synchrony data  *n* = 37 | Dyads with physiological synchrony data  *n* = 57 |  |  |
| --- | --- | --- | --- | --- |
|  | *M (SD)* | *M (SD)* | *t*(df) | *p*-value^1^ |
| Maternal age | 32.26 (4.42) | 32.69 (3.78) | -0.48 (64.24) | .633 |
| Infant age (months) | 3.53 (0.29) | 3.63 (0.34) | -1.55 (85.6) | .124 |
| Maternal BMI | 23.29 (3.48) | 23.24 (2.83) | 0.07 (63.73) | .947 |
| Infant size (cm) | 52.44 (3.12) | 52.04 (2.66) | 0.65 (66.2) | .519 |
| Infant weight (g) | 3595.56 (482.33) | 3538.29 (503.97) | 0.55 (77.07) | .586 |
| EPDS | 2.94 (2.81) | 2.64 (2.60) | 0.53 (70.83) | .600 |
| PBQ | 6.44 (3.74) | 7.43 (4.48) | -1.14 (83.94) | .258 |
| Beh. Synch. (play) | 3.74 (0.83) | 3.69 (0.62) | 0.329 (63.58) | .743 |
| Beh. Synch. (reunion) | 3.36 (0.98) | 3.48 (0.69) | -0.618 (61.22) | .539 |
|  | *N* (%) | *N* (%) | χ²(df) | *p*-value^2^ |
| Infant sex (female) | 19 (51%) | 24 (43%) | 0.647 (1) | 0.421 |

*Note.* Infant age refers to T1 assessment. Infant size and weight at birth.

^1^Welch Two Sample t-test

^2^Chi2-Test

**Figure S1**

*Data processing pipeline*
